# Auditory localization: a comprehensive practical review

**DOI:** 10.3389/fpsyg.2024.1408073

**Published:** 2024-07-10

**Authors:** Alessandro Carlini, Camille Bordeau, Maxime Ambard

**Affiliations:** Laboratory for Research on Learning and Development (LEAD), CNRS UMR, Université de Bourgogne, Dijon, France

**Keywords:** acoustics, auditory localization, ITD, ILD, HRTF, action perception coupling

## Abstract

Auditory localization is a fundamental ability that allows to perceive the spatial location of a sound source in the environment. The present work aims to provide a comprehensive overview of the mechanisms and acoustic cues used by the human perceptual system to achieve such accurate auditory localization. Acoustic cues are derived from the physical properties of sound waves, and many factors allow and influence auditory localization abilities. This review presents the monaural and binaural perceptual mechanisms involved in auditory localization in the three dimensions. Besides the main mechanisms of Interaural Time Difference, Interaural Level Difference and Head Related Transfer Function, secondary important elements such as reverberation and motion, are also analyzed. For each mechanism, the perceptual limits of localization abilities are presented. A section is specifically devoted to reference systems in space, and to the pointing methods used in experimental research. Finally, some cases of misperception and auditory illusion are described. More than a simple description of the perceptual mechanisms underlying localization, this paper is intended to provide also practical information available for experiments and work in the auditory field.

## 1 Introduction

A bird singing in the distance, a friend calling us, a car approaching quickly… our auditory system constantly works to transmit information coming from our surroundings. From exploring our environment to identifying and locating dangers, auditory localization plays a crucial role in our daily lives and fast and accurate auditory localization is of vital importance. However, how does our perceptual system locate the origin of sounds so accurately? This review aims to provide a comprehensive overview of the capabilities and mechanisms of auditory localization in humans. The literature has so far extensively described the fundamental mechanisms of localization, whereas recent findings add new information about the importance of ancillary mechanisms to resolve uncertainty conditions and increase effectiveness. This paper aims to summarize the totality of these factors. Moreover, for the sake of completeness, we have supplemented the review with some practical insights. We enriched the functional description with relevant information about the methods of study, measurement, and perceptual limits.

There is growing interest in auditory localization mechanisms, as they have a great potential for improving the spatialization of sound in emerging immersive technologies, such as virtual reality and 3D cinema. Even more interesting and challenging is their use in sensory augmentation or substitution devices, used to improve the lives of people with perceptual disabilities. This work aims to provide a concise and effective explanation of the relation between the structure of the acoustic signals and the human sound source localization abilities, for both theoretical researches and practical areas. Accordingly, we have omitted an examination of the neural correlates involved in auditory localization. We invite readers interested in this topic to refer to the specific literature.

The body of this review is divided into three sections. In the first part, an overview of the mechanisms involved in human 3D sound localization, as well as the associated capabilities and limitations, is given in order to provide a holistic understanding of the field. In the second part, we provide a more detailed explanation of the auditory cues. Finally, we present other factors that influence the localization of sound source, such as pointing and training methods, and sound characteristics (frequency, intensity…), alterations that can even lead to illusionary phenomena.

## 2 Localizing a sound in space

### 2.1 Auditory localization is based on auditory perception

Auditory localization naturally relies on auditory perception. Its characteristics and limitations are primarily determined by the capabilities of the human perceptual system and exhibit considerable interindividual variability. Although the study of the auditory system has ancient origins, it was not until the 19th century that research started to focus on the functional characteristics of our auditory system, as well as on localization abilities. In the 20th century, the growing knowledge of the perceptual system and the adoption of more rigorous protocols revealed the complexity of the mechanisms of acoustic localization as well as the importance of using appropriate methods of investigation (Grothe and Pecka, 2014; Yost, 2017). Indeed, measures of auditory localization can be influenced by many factors. In experimental tests, for example, participants’ responses depend on the type of auditory stimuli as well as the order in which they are presented, the way the sound spreads through the environment, the age of the listener, and the method used to collect responses (Stevens, 1958; Wickens, 1991; Reinhardt-Rutland, 1995; Heinz et al., 2001; Gelfand, 2017). Results of experimental research also highlight a high level of inter-subject variability that affects responses and performance in various experimental tests (Middlebrooks, 1999a; Mauermann et al., 2004; Röhl and Uppenkamp, 2012).

The cues used by the auditory system for localization are mainly based on the timing, intensity, and frequency of the perceived sound. The perceptual limits of these three quantities naturally play an important role in acoustic localization. Regarding the perception of sound intensity, the threshold varies with frequency. Given a sound of 1 kHz, the minimum pressure difference that the human hearing system can detect is approximately 20 μPa, corresponding by definition to the intensity level of 0 dB SPL (Howard and Angus, 2017). The perceived intensity of a sound does not correspond to the physical intensity of the pressure wave, and the perceptual bias varies depending on the frequency of the sound (Laird et al., 1932; Stevens, 1955). Fletcher and Munson, and Robinson and Dadson successively, carried out the best-known studies concerning the correspondence between physical and perceived sound intensity (Fletcher and Munson, 1933; Robinson and Dadson, 1956). Today, ISO 226:2003 defines the standard auditory equal-loudness level chart. To do this, it uses a protocol based on free-field frontal and central loudspeaker playback to participants aged 18 ÷ 25 years from a variety of countries worldwide. With regard to the spectrum of frequencies audible to the human auditory system, the standard audible range is considered to be between 20 Hz and 20,000 Hz. However, auditory perception depends on many factors, and especially on the age of the listener. Performance is at its highest at the beginning of adulthood, at around 18 years of age, and declines rapidly: by 20 years of age, the upper limit may have dropped to 16 kHz (Howard and Angus, 2017). The reduction is continuous and progressive, mainly affecting the upper threshold. Above the age of 35~40 years, there is a significant reduction in the ability to hear frequencies above 3–4 kHz (Howarth and Shone, 2006; Fitzgibbons and Gordon-Salant, 2010; Dobreva et al., 2011).

Finally, the perceived frequency of sounds does not correspond exactly to their physical frequency but instead shows systematic perceptual deviations. The best-known psychoperceptual scales that relate sound frequency to pitch are the Mel scale (Stevens and Volkmann, 1940), the Bark scale (Zwicker, 1961), and the ERB scale (Glasberg and Moore, 1990; Moore and Glasberg, 1996).

### 2.2 Main characteristics of localization

Auditory localization involves several specialized and complementary mechanisms. Four main types of cue are usually mentioned: the two binaural cues (interaural time and level differences), monaural spectral cues due to the shading of the sound wave by the listener body, and additional factors such as reverberation or relative motion that make localization more effective – and more complex to study. These mechanisms operate simultaneously or complementarily in order to compensate for weaknesses in any of the individual mechanisms, resulting in high accuracy over a wide range of frequencies (Hartmann et al., 2016). In this section, we provide an overview of the main mechanisms that are explained in more detailed in the second and third sections.

The main binaural cues that our perceptual system uses to localize sound sources more precisely are the Interaural Time Difference (ITD) and the Interaural Level Difference (ILD) (mechanisms based on differences in time and intensity, respectively, as described below). At the beginning of the last century, Lord Rayleigh proposed the existence of two different mechanisms, one operating at low frequencies and the other at high frequencies. This is known as the Duplex Theory of binaural hearing. Stevens and Newman found that localization performances are best for frequencies below about 1.5 kHz and above about 5 kHz (Rayleigh, 1907; Stevens and Newman, 1936). The smallest still perceivable interaural difference between our ears is about 10 μs for ITD, and about 1 dB for ILD (Mills, 1958; Brughera et al., 2013; Gelfand, 2017).

The localization of a sound source in space is characterized by a certain amount of uncertainty and bias, which result in estimation errors that can be measured as constant error (accuracy) and random error (precision). The type and magnitude of estimation errors depend on the properties of the emitted sound, the characteristics of the surroundings, the specific localization task, and the listener’s abilities (Letowski and Letowski, 2011). Bruns and colleagues investigated two methods (error-based and regression-based) for calculating accuracy and precision. The authors pointed out that accuracy and precision measures, while theoretically distinct in the two paradigms, can be strongly correlated in experimental datasets (Bruns et al., 2024). Garcia and colleagues proposed a comparative localization study, comparing performance before and after training. Their results show that both constant errors and variability in auditory localization tend to increase when auditory uncertainty increases. Moreover, such biases can be reduced through training with visual feedback (Garcia et al., 2017).

As we will see below, sound source localization is more accurate in the horizontal plane (azimuth) than in the vertical plane (elevation). Localization performances in the third dimension (distance) are less accurate than for either azimuth or elevation and are subject to considerable inter-subject variability (Letowski and Letowski, 2011). Moreover, these abilities change with the age of the listener. Dobreva and colleagues found that young subjects systematically overestimate (overshoot) horizontal position and systematically underestimate vertical position. Moreover, the magnitude of the effect varies with the sound frequency. In middle-aged subjects, these authors found a pronounced reduction in the precision of horizontal localization for narrow-band targets in the range 1,250 ÷ 1,575 Hz. Finally, in elderly subjects, they found a generalized reduction in localization performance in terms of both accuracy and precision (Dobreva et al., 2011). Otte and colleagues also performed a comparative study of localization abilities, testing three different age groups ranging from 7 to 80 years. Their results are somewhat more positive, especially for the older age group: localization ability remains fully effective, even in the early phase of hearing loss. Interestingly, they also found that older adults with big ears had significantly better elevation localization abilities. This advantage does not appear in azimuth localization. Young subjects, with smaller ears, require higher frequencies (above 11 kHz) to accurately localize the elevation of sounds (Otte et al., 2013).

The quantitative evaluation of human performance is based on two types of localization estimation: Absolute localization (a sound source must be localized directly, usually with respect to a listener-centered reference system), and Discrimination (two sound sources have to be distinguished in the auditory signal, either simultaneously or sequentially).

Concerning absolute localization, in frontal position, peak accuracy is observed at 1÷2 degrees for localization in the horizontal plane and 3÷4 degrees for localization in the vertical plane (Makous and Middlebrooks, 1990; Grothe et al., 2010; Tabry et al., 2013). Investigating the frontal half-space, Rhodes – as well as Tabry and colleagues more recently – found that the azimuth and the elevation error grows linearly as the distance of the target from the central position increases. Using the head-pointing method, Tabry et al. found that the error grows up to ~20 degrees for an azimuth of ±90° and up to ~30 degrees for an elevation of −45° and +67° (Rhodes, 1987; Tabry et al., 2013).

Among the discrimination paradigms, the most commonly used is the Minimal Audible Angle (MAA), which is defined as the smallest angle that a listener can discriminate between two successively presented stationary sound sources. Mills developed the MAA paradigm and studied the human ability to discriminate lateralization (azimuth). He showed that the MAA threshold also depends on the frequency of the sound and found that MAA performance is better for frequencies below 1,500 Hz, and above 2,000 Hz. The best performance is obtained in the frontal field with an MAA accuracy in the frontal-central position equal to 1 ÷ 2 degrees in azimuth. In a more recent study, Aggius-Vella and colleagues found slightly larger values: they reported an MAA threshold in azimuth of 3° in frontal position, and 5° in rear position. The above values refer to sources positioned at ear level. When they moved the sound source to foot level, Aggius-Vella and colleagues found an MAA threshold of 3° in both front and rear positions. In a more recent study, Aggius-Vella and colleagues placed the sound source 1 m above the floor and found an MAA threshold of 6° in the front position and 7° in the rear position. It is important to note that the works of Mills and Aggius-Vella used two different protocols: while Mills used audio headphones to play the sound, Aggius-Vella and colleagues used a set of aligned loudspeakers (Mills, 1958, 1960; Mills and Tobias, 1972; Aggius-Vella et al., 2018, 2020). Similarly, a discrimination paradigm known as MADD (Minimal Audible Distance Discrimination) is used for the distance dimension. Using a MADD-type paradigm, Aggius-Vella et al. (2022) reported better distance discrimination abilities in the front space (19 cm) than in the rear space (21 cm). They found a comparable effect of the spatial region using a distance bisection paradigm, which revealed a lower threshold (15 cm) in the front space than in the rear space (20 cm) (Aggius-Vella et al., 2022). It is also relevant to note that some authors have criticized the MAA paradigm, claiming that the experimental protocol enables responses to be produced based on criteria other than relative discrimination through the use of identification strategies (Hartmann and Rakerd, 1989a).

A second, and important, discrimination paradigm is the CMAA (Concurrent Minimum Audible Angle), which measures the ability to discriminate between two simultaneous stimuli. In the frontal position, Perrott found a CMAA threshold of 4°÷10° (Perrott, 1984). Brungart and colleagues investigated the discrimination and localization capabilities of our auditory system when faced with multiple sources (up to 14 tonal sounds) with or without allowed head movement. They found that although localization accuracy systematically decreased as the number of concurrent sources increased, overall localization accuracy was nevertheless still above chance even in an environment with 14 concurrent sound sources. Interestingly, when there are more than five simultaneous sound sources, exploratory head movements cease to be effective in improving localization accuracy (Brungart et al., 2005). Zhong and Yost found that the maximum number of simultaneous separate stimuli that our perceptual system can easily discriminate is approximately 3 for tonal stimuli and 4 for speech stimuli (Zhong and Yost, 2017), which is in line with many studies that have shown that localization accuracy is significantly improved when localizing broadband sounds (Butler, 1986; Makous and Middlebrooks, 1990; Wightman and Kistler, 1992, 1997; Gelfand, 2017).

### 2.3 Reference system and localization performances

Each target in space is localized according to a reference system. In the case of human perception, experimental research suggests that our brain uses different reference systems, both egocentric and allocentric, and is able to switch easily between them (Graziano, 2001; Wang, 2007; Galati et al., 2010). More specifically, with regard to auditory perception, Majdak and colleagues describe the different mechanisms involved in the creation of the internal representation of space (Majdak et al., 2020). Moreover, research works such as those of Aggius-Vella and Viaud-Delmon also show that these mechanisms are closely related to other perceptual channels, and in particular the visual and sensorimotor channels, making it possible to calibrate the reference system more accurately and improve the spatial representation (Viaud-Delmon and Warusfel, 2014; Aggius-Vella et al., 2018).

With regard to the experimental protocols used in the field of auditory localization, almost all research works have adopted a reference system centered on the listener, generally positioning the origin at the midpoint of the segment joining the two ears (Middlebrooks et al., 1989; Macpherson and Middlebrooks, 2000; Letowski and Letowski, 2011). In contrast, some research has used an allocentric reference system in which the positions of the localized sound sources have to be reported with reference to a fictional head (tangible or digital) that represents that of the participant (tangible: Begault et al., 2001; Pernaux et al., 2003; Schoeffler et al., 2014) (digital: Gilkey et al., 1995).

The reference system and the pointing system used to provide the response are closely related. The use of an egocentric reference system is usually preferred because it prevents participants from making projection errors when giving responses. For example, Djelani and colleagues demonstrated that the God’s Eye Localization Pointing (GELP) technique, that is an allocentric reference-and-response system where the perceived direction of the sound is indicated by pointing at a 20 cm diameter spherical model of auditory space, brings about certain systematic errors as a consequence of the projection from the participant’s head to its external representation (Djelani et al., 2000). Similarly, head or eye pointing is preferred since it avoids the parallax errors that frequently occur with pointing devices. In addition, many authors prefer to use head or gaze orientation as a pointing system, because it is considered more ecological and does not require training or habituation (Makous and Middlebrooks, 1990; Populin, 2008).

**Figure 1:**
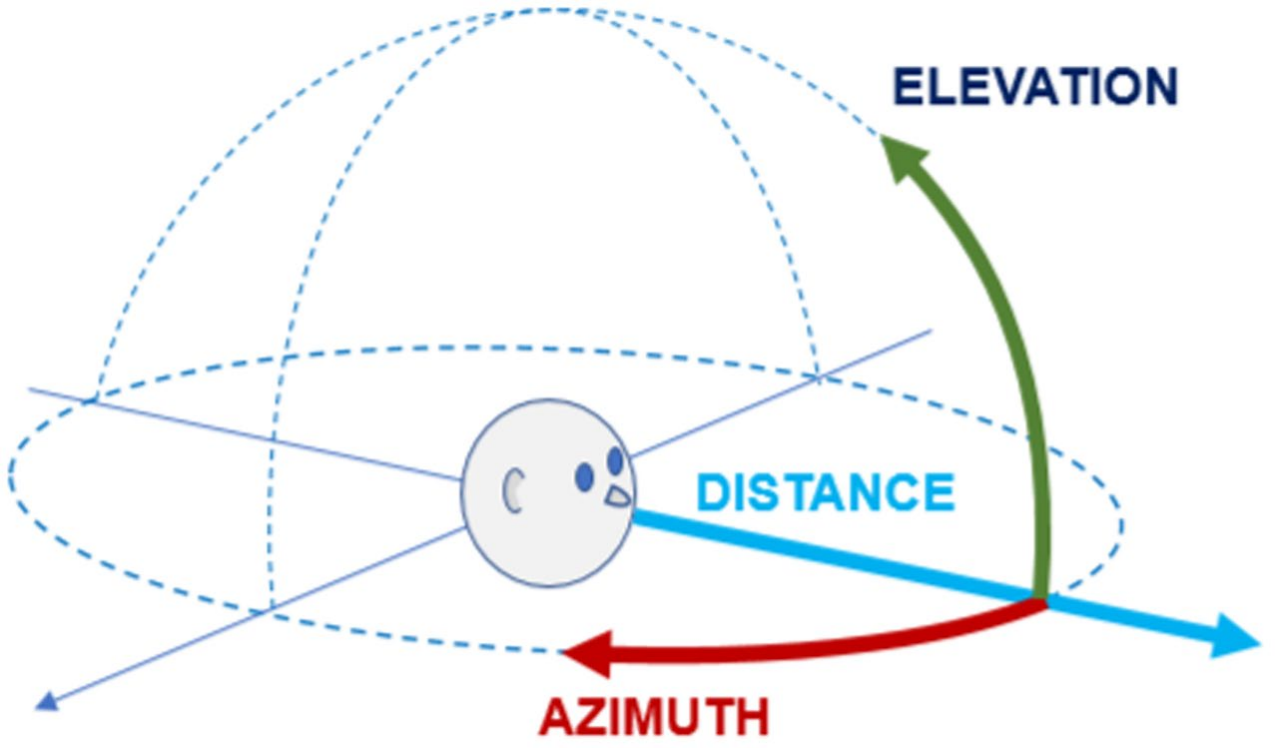
Reference system. The most commonly used reference system for locating a sound source in three-dimensional space is the polar coordinate system. The reference system is centered on the listener and divides space according to two angular coordinates (azimuth in the horizontal plane, elevation in the vertical plane) and one linear coordinate (distance or depth), as shown in the figure.

The most commonly used reference system in studies on spatial hearing is the bipolar spherical coordinate system (Figure 1). This coordinate system consists of two angular dimensions, θ (azimuth or declination) and φ (elevation), and one linear dimension, d (distance or depth) (Middlebrooks, 1999b; McIntyre et al., 2000; Jerath et al., 2015). In some cases, a cylindrical system (in which the angular elevation is replaced by a linear elevation parameter) (Febretti et al., 2013; Sherlock et al., 2021), or a Cartesian system (Parise et al., 2012) is preferred. An alternative reference system is the Interaural-polar coordinate system, which has been described by Majdak as corresponding more closely to the human perceptual system and consists of a lateral angle α, a polar angle β, and a linear distance *r* (Majdak et al., 2020).

#### 2.3.1 Azimuth

In spherical coordinate systems, the Azimuth is defined as the angle between the projection of the target position on the horizontal plane and a reference meridian, measured from above either clockwise (Rychtáriková et al., 2011; Parseihian et al., 2014) or, less commonly, counter-clockwise (Bronkhorst, 1995; Werner et al., 2016). The standard “zero” reference meridian is the frontal meridian (Vliegen and Van Opstal, 2004; Risoud et al., 2020). Starting from the reference meridian, the horizontal plane is then indexed on a continuous scale of 360 degrees (Iwaya et al., 2003; Oberem et al., 2020) or divided into two half-spaces of 180 degrees, i.e., left and right, with the left half-space having negative values (Makous and Middlebrooks, 1990; Boyer et al., 2013; Aggius-Vella et al., 2020). Conveniently, the horizontal plane can be also simply divided into front and rear (or back) half-spaces.

The most important cues for auditory localization in the azimuthal plane are the ILD and ITD. However, the effectiveness of ILD and ITD is subject to some limitations relating to the frequency of the sound, and other mono-or bin-aural strategies are required to resolve ambiguous conditions (see section ILD and ITD) (Van Wanrooij and Van Opstal, 2004).

The best localization performance in the azimuthal plane is found at about 1-2 degrees, namely in the frontal area approximately at the intersection with the sagittal plane (Makous and Middlebrooks, 1990; Perrott and Saberi, 1990).

#### 2.3.2 Elevation

In the spherical coordinate system, the Elevation (or polar angle) is the angle between the projection of the target position on a vertical frontal plane and a zero-elevation reference vector. This is commonly represented by the intersection of the vertical frontal plane with the Azimuth plane, with positive values being assigned to the upper half-space and negative values to the lower half-space, thus obtaining a continuous scale [−90°, +90°] (Figure 1; Middlebrooks, 1999b; Trapeau and Schönwiesner, 2018; Rajendran and Gamper, 2019). Occasionally, the zero-elevation reference is assigned to the Zenith and the maximum value is assigned to the Nadir, resulting in a measurement scale consisting only of positive values [0°, +180°] (Oberem et al., 2020).

Elevation estimation relies primarily on monaural spectral cues, mainly resulting from the interaction of the sound with the auricle. These interactions cause modulations of the sound spectrum reaching the eardrum and are grouped together under the term Head-Related Transfer Functions (HRTF), see HRTF section (Ahveninen et al., 2014; Rajendran and Gamper, 2019). Otte et al. (2013) graphically show the variation of the sound spectrum as a function of both elevation and the various individual anatomies of the outer ear. Auditory localization in the vertical plane has lower spatial resolution than that in the horizontal plane. The best localization performance in terms of elevation is of the order of 4-5 degrees (Makous and Middlebrooks, 1990).

#### 2.3.3 Distance

In acoustic localization, distance is simply defined as the linear measure of the conjunction between the midpoint of the segment joining the two ears and the sound source. The human auditory system can use multiple acoustic cues to estimate the distance of a sound source. The two main strategies for estimating the distance from a sound source are both based on the acoustic intensity of the sound reaching the listener. The first is based on an evaluation of the absolute intensity of the direct wave. The second, called the Direct-to-Reverberant energy Ratio “DRR,” is based on a comparison between the direct wave and the reverberated sound waves (Bronkhorst and Houtgast, 1999; Zahorik, 2002; Guo et al., 2019). In addition, other cues, such as familiarity with the source or the sound, the relative motion between listener and source, and spectral modifications, provide important indications for distance estimation (Little et al., 1992). Prior knowledge of the sound and its spectral content plays a role in the ability to correctly estimate the distance (Neuhoff, 2004; Demirkaplan and Haclhabibogˇlu, 2020). Some studies have suggested that listeners may also use binaural cues to determine the distance of sound, especially if the sound source is close to the side of the listener’s head. These strategies are thought to use the ITD to localize the azimuth and the ILD to estimate the distance. Given the limitations of the ILD, these strategies would only be effective for distances less than 1 meter (Bronkhorst and Houtgast, 1999; Kopčo and Shinn-Cunningham, 2011; Ronsse and Wang, 2012). Generally speaking, the accuracy of distance estimation varies with the magnitude of the distance itself. Distance judgments are generally most accurate for sound sources approximately 1 m from the listener. Closer distances tend to be overestimated, while greater distances are generally underestimated (Fontana and Rocchesso, 2008; Kearney et al., 2012; Parseihian et al., 2014). For distant sources, the magnitude of the error increases with the distance (Brungart et al., 1999).

## 3 Auditory cues for sound source localization

Sound localization is based on monaural and binaural cues. Monaural cues are processed individually in one ear, mostly providing information that is useful for vertical and antero-posterior localization. Binaural cues, by contrast, result from the comparison of sounds reaching the two ears, and essentially provide information about the azimuth position of the sound source. The sections below explore these localization mechanisms.

### 3.1 ITD and IPD

**Figure 2:**
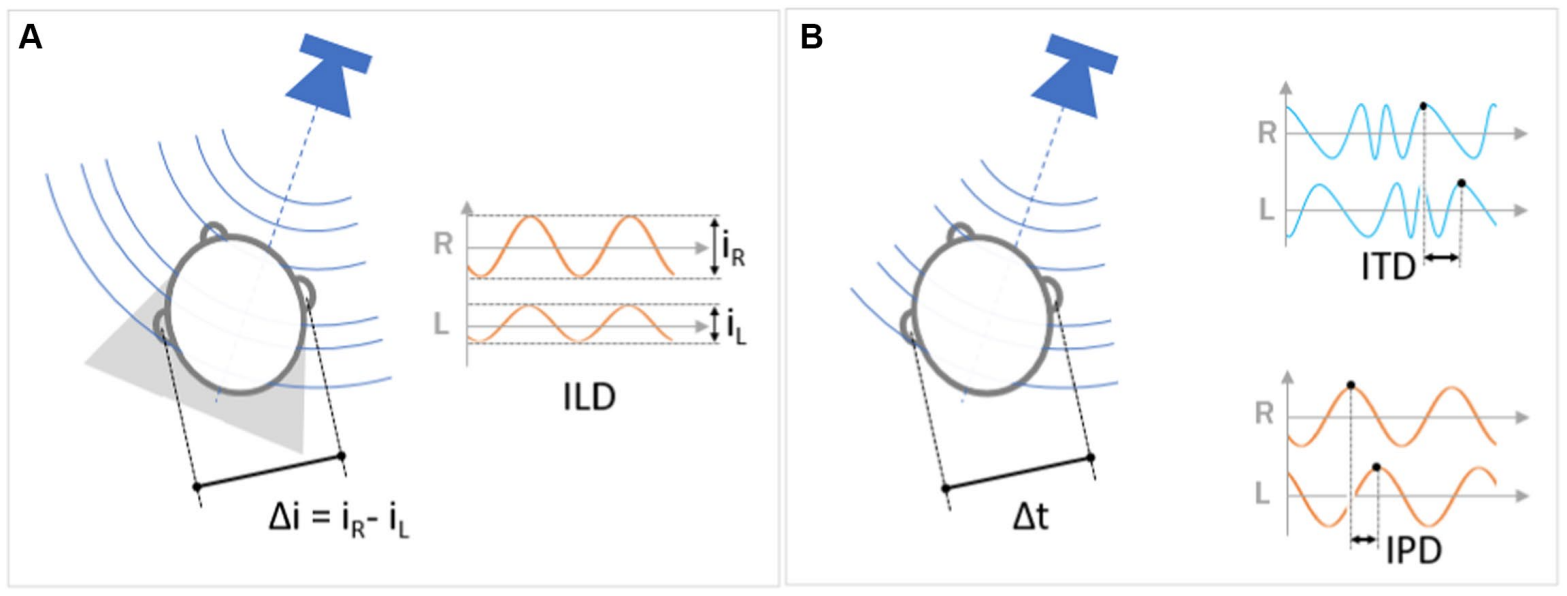
ILD, ITD and IPD. The fundamental binaural cues for auditory localization are based on the difference in perception between the two ears in terms of both intensity and time. A source located in front of the listener produces a sound wave that arrives at both ears identically (the direct wave arrives at the same time and with the same intensity). By contrast, a lateral source results in a difference in signal intensity between the right and left ears, respectively i_R_ and i_L_ (Δi, Panel **A**), and in arrival time (Δt, Panel **B**). **(A)** In the case of a lateral source, the sound stimulus arriving at the more distant ear is less intense, due to its greater distance from the source and the shadow effect produced by the head itself. Interaural Level Difference (“ILD”) is the perceptual mechanism that estimates the position of the source as a function of the intensity difference between the two ears. **(B)** The ear more distant from the source receives the sound with a time delay. The Interaural Time Differences (“ITD”) is the perceptual mechanism for localizing the sound source based on the time delay between the two ears. Fine variations in azimuth localization are also measured as Interaural Phase Differences (“IPD”), based on the phase differences between the waves reaching each ear.

Let us consider a sound coming, for instance, from the right side of the head: it reaches the right ear before the left ear. The difference in reception times between the two ears is called the Interaural Time Difference (ITD). It constitutes the dominant cue in estimating the azimuth of sound sources at frequencies below 1,500 Hz and loses its effectiveness at higher frequencies. ITD is actually related to two distinct processes for measuring the asynchrony between the acoustic signals received by the left and the right ears. The first process measures the temporal asynchrony of the onset between the two sounds reaching the left and right ear or between distinctive features that serve as a reference, such as variations. The second process measures the phase difference between the two sound waves reaching each ear, which represents an indirect measure of the temporal asynchrony. We refer to this second mechanism as the Interaural Phase Difference (IPD). Panel B of Figure 2 represents the two processes in graphic form.

The smallest detectable interaural time difference (i.e., the maximum ITD sensitivity) is in the order of 10 μs, both for noise or complex stimuli (9 μs) (Klumpp and Eady, 1956; Mills, 1958) and for pure tones (11 μs) (Klumpp and Eady, 1956; Brughera et al., 2013). More recently, Thavam and Dietz found a larger value with untrained listeners (18.1 μs), and a smaller value with trained listeners (6.9 μs), using a band-pass noise of 20–1,400 Hz at 70 dB (Thavam and Dietz, 2019). By contrast, the largest ITD is of the order of 660–790 μs and corresponds to the case of a sound generated in front of one ear (Middlebrooks, 1999a; Gelfand, 2017). For instance, considering the spherical model of a human head with radius Rh = 8.75 cm combined with a sound speed *Vs* = 34,300 cm/s (at 20°C), we obtain an ITD threshold value = (3*Rh/*Vs*)*sin(90°) = 765.3 μs (Hartmann and Macaulay, 2014).

Tests reveal that the best azimuth localization performances using only ITD/IPD are obtained with a 1,000 Hz sound, allowing an accuracy of 3~4 degrees (Carlile et al., 1997). Beyond this frequency, ITD/IPD rapidly lose effectiveness due to the relationship between the wavelength of the sound and the physical distance between the listener’s ears. Early research identified the upper threshold value at which ITD loses its effectiveness at between 1,300 Hz and 1,500 Hz (Klumpp and Eady, 1956; Zwislocki and Feldman, 1956; Mills, 1958; Nordmark, 1976). Most recent research has found residual efficacy for some participants at 1,400 Hz and a generalized complete loss of efficacy at 1,450 Hz (Brughera et al., 2013; Risoud et al., 2018).

Due to the cyclic nature of the sound signals, an IPD value for a given frequency can be encountered for multiple azimuth positions. In such cases, the information from the IPD becomes ambiguous and can easily lead to an incorrect azimuth estimation, especially with pure tones (Rayleigh, 1907; Bernstein and Trahiotis, 1985; Hartmann et al., 2013). Various different azimuthal positions may appear indistinguishable by IPD because the phase difference is equal to a multiple of the wavelength (Elpern and Naunton, 1964; Sayers, 1964; Yost, 1981; Hartmann and Rakerd, 1989a). The quantity and angular values of these ambiguous directions depend on the wavelength of the sound: the higher the frequency of the sound, the greater the number of ambiguous positions generated. Consequently, the ITD/IPD operates more effectively at low frequencies.

### 3.2 ILD

When a sound source is positioned to the side of the head, one of the ears is more exposed to it. The presence of the head produces a shadowing effect on the sound in the direction of propagation (sometimes referred to as HSE – head-shadow effect). As a result, the sound intensity (or “level”) at the ear shadowed by the head is lower than at the opposite ear (see Panel A of Figure 2). The amount of shadowing depends on the angle, frequency and distance of the sound as well as on individual anatomical features. Computing the difference in intensity between the two ears provides the auditory cue named Interaural Level Difference (ILD). ILD is zero for sounds originating in the listener’s sagittal plane, while for lateral sound sources it increases approximately proportionally to the sine of the azimuth angle (Mills, 1960). From a physical point of view, the head acts as an obstacle to sound propagation for wavelengths shorter than the head size. For longer wavelengths (i.e., lower frequency), however, the sound wave passes relatively easily around the head and the difference in intensity of the soundwaves reaching the two ears becomes imperceptible. Consequently, sound frequencies higher than 4,000 Hz are highly attenuated and the ILD is a robust cue for azimuth estimation, whereas for frequencies lower than 1,000 Hz, the ILD becomes completely ineffective (Shaw, 1974).

In a reverberant environment, as the distance from the sound source increases, the sound waves reflect off multiple surfaces, resulting in a more complex received binaural signal. This leads to fluctuations in the Interaural Level Differences (ILDs), which have been shown to affect the externalization of sound (the perception that the sound is located at a distance from the listener’s head) (Catic et al., 2013).

### 3.3 Limits of ITD and ILD

**Figure 3:**
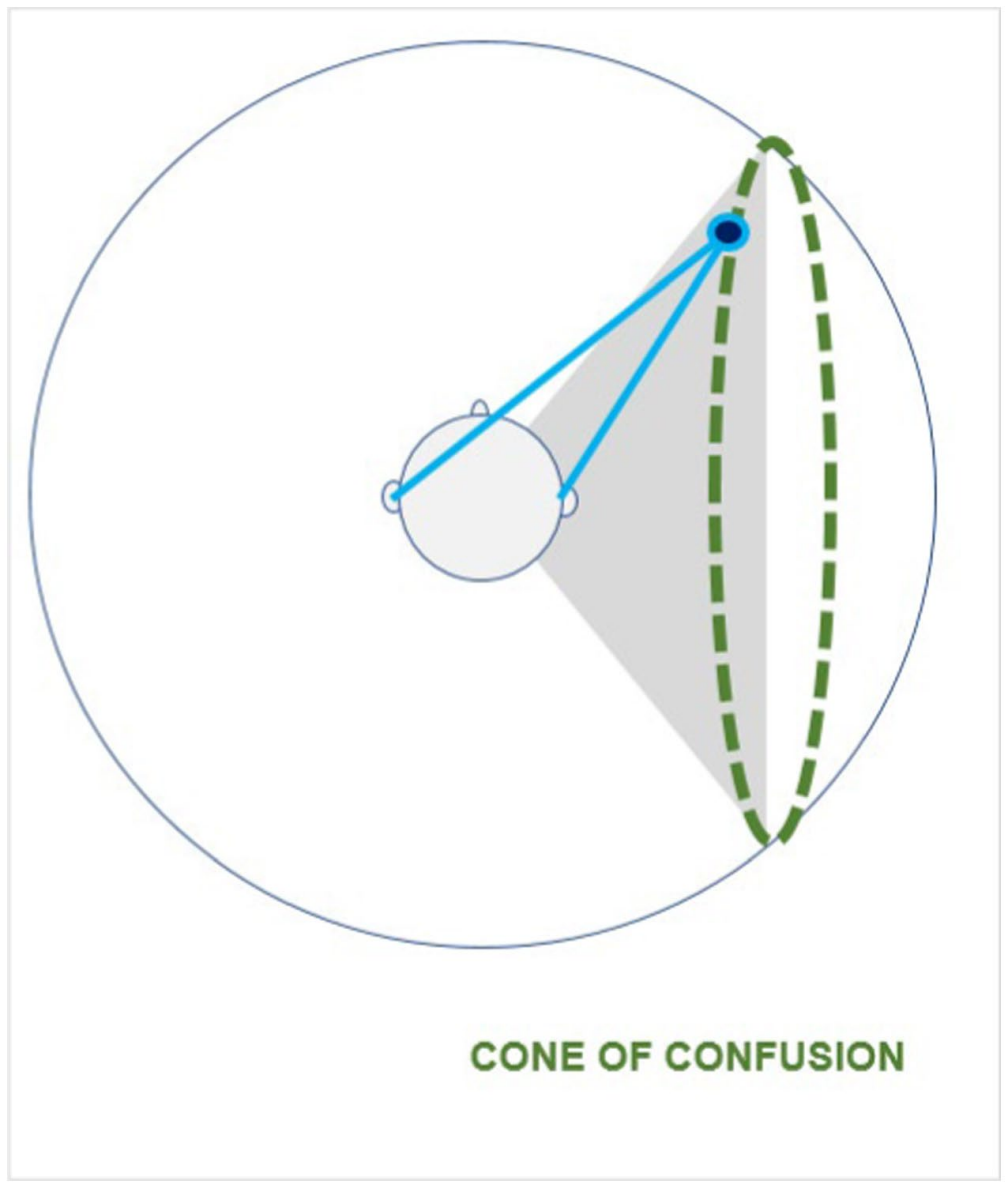
Cone of confusion. A sound emitted from any point on the dotted line will give rise to the same ITD because the difference between the distances to the ears is constant. This set of points forms the “cone of confusion.”

ITD and ILD appear to be two complementary mechanisms, the former being optimized for low frequencies and the latter for high frequencies. Therefore, our acoustic system exhibits the poorest performance in terms of acoustic localization in the range between 1,500 Hz and 4,000 Hz (approximately) (Yost, 2016; Risoud et al., 2018). However, given a spherical head shape, even a perfect determination of the ILD or the ITD would not be sufficient to permit complete and unambiguous pure tone localization. The ITD depends on the difference between the distances from the sound source to each of the two ears, and the ILD depends on the angle of incidence of the sound wave relative to the axis of the ears. Thus, every point situated at the same distance and the same angle of incidence would theoretically result in the same ITD and ILD. Mathematically, the solution to both systems is not a single point, but a set of points located on a hyperbolic surface, whose axis coincides with the axis of the ears. This set of points, for which the difference in distance to the two ears is constant, is called the “cone of confusion” (Figure 3). More information is required in order to obtain an unambiguous localization of the sound source. Additional factors such as reverberation, head movement, and a wider sound bandwidth greatly reduce the uncertainty of localization. In ecological conditions with complex sounds, this type of uncertainty is mainly resolved by analyzing the frequency modulation produced by the reverberation of the sound wave at the outer ear, head and shoulders: the Head-Related Transfer Function.

### 3.4 Head-related transfer function (HRTF)

**Figure 4:**
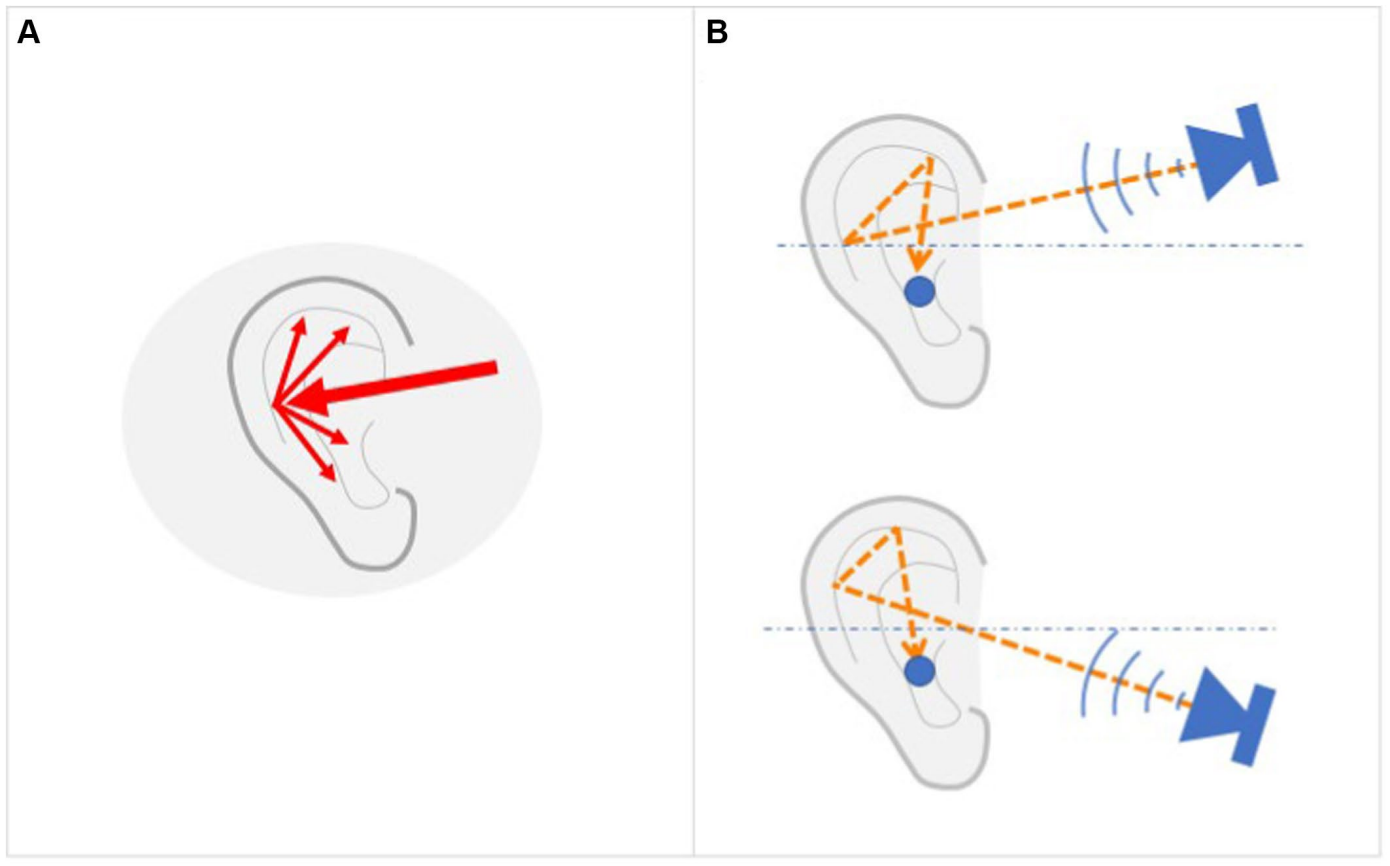
HRTF. **(A)** Arrival of a sound wave at the outer ear and the generation of a series of secondary waves due to reflection in the auricle. **(B)** Each sound wave that reaches the ear thus generates a different set of reflected waves, depending on its original orientation. Using this relationship, our auditory system is able to reconstruct the origin of the sound by analyzing the set of waves that reach the eardrum.

Our perceptual system has evolved with a special ability to decode the complex structure of the sounds reaching our ears, thus enabling us to estimate the spatial origin of sounds. Under ecological conditions, each eardrum receives not only the direct sound wave of each sound that reaches the listener’s ear but also a complex series of sound waves reflected from the shoulders, head, and auricle (Figure 4). This complex set of new waves that depend on the orientations of the head and the torso relatively to the sound source, greatly enriches the spatial information contained in and carried by the sound. These reflected waves are used by the auditory system to extract spatial information and to infer the origin of the sound. This acoustic filtering can be characterized by transfer functions called the Head-Related Transfer Functions (HRTFs). HRTFs are considered monaural cues because the spectral distortions they produce depend solely on the position of the sound source relative to the orientation of the body, the head, and the ear. No comparison between the signals received by both ears is required.

Several studies have reported better HRTF localization performance for sound sources positioned laterally than for sources positioned frontally and rearwardly. For example, Mendonça, and later Oberem, found an improvement in lateral localization ranging from a few degrees to ten degrees, depending on the test conditions (Wightman and Kistler, 1989; Mendonça et al., 2012; Oberem et al., 2020). However, a marked interindividual variability in localization performances as well as in the ability and time required to adapt to non-individualized HRTFs has also been observed (Mendonça et al., 2012; Stitt et al., 2019). Begault and colleagues conducted a study on the localization of speech stimuli in which they compared individualized and non-individualized HRTFs (obtained from a dummy head). One of the aims of the research was to assess whether the relationship between listener and dummy head size was a predictor of localization errors. Contrary to initial expectations, the results showed no correlation between localization error and head size difference (Begault et al., 2001). Another interesting and rather unexpected result reported by both Begault et al. and Møller et al. was that individualized HRTFs do not bring about an advantage in speech localization accuracy compared to non-individualized HRTFs. To explain this finding, Begault and colleagues suggest that most of the spectral energy of speech is in a frequency range in which ITD cues are more prominent than HRTF spectral cues (Møller et al., 1996; Begault et al., 2001).

The way in which the sound is modified by the reflection in the outer ear and the upper body can be recorded experimentally and reproduced by transfer functions. The corresponding information can be used in practice to play sounds through headphones and create the perception that each sound is coming from a distant desired origin, thus creating a three-dimensional virtual auditory environment (Wightman and Kistler, 1989; Møller, 1992). Nowadays, HRTFs are the most frequent way of creating acoustic spatialization systems, strongly driven by the demand for higher-performance entertainment systems, games, and specially augmented/virtual reality systems (Begault, 2000; Poirier-Quinot and Katz, 2018; Geronazzo et al., 2019; Andersen et al., 2021). Because everyone’s anatomy is different and ear shapes are very individual, HRTF techniques can be divided into two main categories depending on whether they use individualized or non-individualized transforms. Although special environments and extensive calibrations are needed in order to obtain individualized transforms, they do, however, permit more accurate auditory spatial perception (Pralong and Carlile, 1996; Meshram et al., 2014; Gan et al., 2017). Individualized HRTFs also require interpolation techniques, as HRTFs are typically measured at discrete locations in space (Freeland et al., 2002; Grijalva et al., 2017; Acosta et al., 2020). Conversely, non-individualized HRTFs are generic HRTFs, obtained on the basis of averaged or shared parameters, which are then universally applied. They are easier to obtain, but are known to cause spatial discrepancies such as poor externalization, elevation errors, misperception, and front-back confusion (Wenzel et al., 1993; Begault, 2000; Berger et al., 2018).

Various methods have been developed to generate individualized HRTF based on anthropometric data: by analytically solving the interaction of the sound wave with the auricle (Zotkin et al., 2003; Zhang et al., 2011; Spagnol, 2020), using photogrammetry (Mäkivirta et al., 2020), or based on deep-learning neural networks (Chun et al., 2017; Lee and Kim, 2018; Miccini and Spagnol, 2020). At the same time, several studies have also investigated the possibility of using a training phase to improve the effectiveness of non-individualized HRTFs. Stitt and colleagues found a positive effect of training (Stitt et al., 2019). Mendonça and colleagues investigated whether feedback is necessary in the training phase. Their results clearly indicate that simple exposure to the HRTF sounds without feedback does not produce a significant improvement in acoustic localization (Mendonça et al., 2012).

### 3.5 Reverberation

Reverberation enriches the sound along its path with additional information concerning the environment, the sound itself, and its source. Under anechoic conditions, the listener estimates the direction and distance of the sound source based on its intensity and the spectral content of the sound. When reverberation is present, however, it provides additional cues for direction and distance estimation, thereby potentially improving localization accuracy. In fact, due to reverberation, successive waves resulting from the reflection of the sound on the surfaces and objects in the environment are added to the direct train of sound waves, acquiring and conveying information about the size and the nature of these surfaces as well as their positions relative to the sound source (Gardner, 1995).

A listener who can move its head is better able to utilize the beneficial effects of reverberation. However, under certain conditions, such as in environments with high levels of reverberation or in the Franssen effect, reverberation can negatively impact localization accuracy (Hartmann and Rakerd, 1989b; Giguere and Abel, 1993).

#### 3.5.1 Reverberation and estimation of azimuth and elevation

In the presence of reverberation, the ITD and ILD must process both the direct wave and the trains of reflected waves, which may come from directions very different from the original direction of the sound. Although reverberation adds a great deal of complexity to auditory percepts, our nervous system has developed the ability to decode the different overlapping pieces of information. A very effective solution for localization in this context is based on the Precedence Effect. As mentioned, when a sound is emitted from a given source, our auditory system first receives the direct sound wave and then, at very short time intervals, sound waves reflected from various surfaces in the surrounding environment. The Precedence effect is a mechanism by which our brain is able to ignore successive reflections and correctly localize the source of sound based on the arrival of the direct sound wave. This mechanism is crucial in supporting localization in echogenic environments (Blauert, 1996; Hartmann, 1999; Nilsson and Schenkman, 2016).

The literature reports conflicting results concerning the effect of reverberation on localization accuracy in terms of the estimation of azimuth and elevation. In a perceptual study in a reverberant room, Hartmann reported a degradation of azimuth localization due to the presence of reverberation (Hartmann, 1983).

Begault and colleagues, on the other hand, found a significant improvement in azimuth localization (of about 5°) in the presence of reverberation, although for some participants the improvement in accuracy was achieved only when head motion was allowed. However, they also found an increase in the average elevation error from 17.6° without reverberation to 28.7° with reverberation (Begault et al., 2001). Conversely, Guski compared the no-reverberation condition with the reverberation condition in which the sound reverberated from a single surface in different orientations. His results showed an overall increase in correct localizations with a sound-reflecting surface on the floor, especially in terms of elevation (Guski, 1990).

#### 3.5.2 Reverberation and distance estimation

Reverberation has proven to be a useful aid when estimating the distance from a sound source. The reverberant wave train is reflected off surfaces, walls and objects and this causes its energy to remain nearly constant over distance – especially indoors. Under ideal conditions, direct propagation in air causes the direct sound wave to lose 6 dB of intensity for every doubling of distance. In a study conducted in a small auditorium, Zahorik demonstrated that the intensity of reflected waves, while being smaller than the direct wave, decreases by only 1 dB for each doubling of distance (Zahorik, 2002). As a result, the ratio between the direct-wave energy and the reflected-wave energy (called the Direct-to-Reverberant Energy Ratio, or DRR) decreases as the distance from the source increases, and has been shown to be a useful perceptual cue for distance estimation (von Békésy, 1938; Mershon and King, 1975; Bronkhorst and Houtgast, 1999).

#### 3.5.3 Reverberation and front-back confusion

Front-back (and back-front) confusion refers to the misperception of a sound position, with the sound being perceived in the wrong hemifield (front or back). This perceptual confusion is particularly common when synthetic sounds are played or audio headphones are used (i.e., in particular when non-individualized HRTFs are used) (Begault et al., 2001; Rychtáriková et al., 2011). It is particularly critical when bone-conduction audio headphones are used since these, by exploiting an alternative communication channel to the inner ear, completely bypass the outer ear and its contribution to spatial perception (Wang et al., 2022). One way to reduce front-back confusion could be to introduce reverberations in synthesized signals. However, the experimental results are ambiguous. Some studies, such as Begault et al. (2001), find that reverberation does not significantly reduce front-back confusion (Begault et al., 2001), while other studies have found that the presence of acoustic reverberation waves significantly improves antero-posterior localization and reduces front-back confusion (Reed and Maher, 2009; Rychtáriková et al., 2011).

#### 3.5.4 Reverberation and sound externalization

The presence of reverberation significantly improves the perceived externalization of sound. Externalization refers to the perception of sound as external to and distant from the listener. Poor externalization causes the listener to perceive sound as being diffused “inside his/her head” and is a typical problem when sound is played through headphones (Blauert, 1997). The three factors known to contribute the most to effective externalization are the use of individualized HRTFs, the relative motion between source and listener, and sound reverberation. When creating artificial sound environments, the addition of reverberation – thus reproducing the diffusion conditions found in the real environment – significantly increases the externalization of the sound, giving the listener a more realistic experience (Zotkin et al., 2002, 2004; Reed and Maher, 2009). Reverberation positively influences the externalization of sounds such as noise and speech (Begault et al., 2001; Catic et al., 2015; Best et al., 2020), including in the case of the hearing aids used by hearing-impaired people (Kates and Arehart, 2018). In some cases, the “early” reflections are sufficient to produce a significant effect (Begault, 1992; Durlach et al., 1992).

### 3.6 Action – perception coupling

Auditory perception in everyday life is strongly related to movement and active information-seeking. The gesture of “lending an ear” is probably the simplest example of action in the service of auditory perception. Experimental research has shown that our auditory system localizes sounds more accurately in two areas: in front of the listener (i.e., 0° azimuth, 0° elevation) and laterally to the listener, i.e., in front of each ear (i.e., ±90° azimuth, 0° elevation). The first position permits the most accurate ITD and ILD-based localization (Makous and Middlebrooks, 1990; Brungart et al., 1999; Tabry et al., 2013), while the second guarantees the highest accuracy that can be obtained on the basis of the HRTF and maximizes the intensity of the sound reaching the eardrum (Mendonça et al., 2012; Oberem et al., 2020).

Unlike some animal species, the human auricle does not have the ability to move independently. As a result, listeners are obliged to move their heads in order to orient their ears. These movements allow them to align the sound in a way that creates the most favorable angle for perception. It should be noted that head movements are strongly related to the orientation of the different senses mobilized, and the resulting movement strategy can be remarkably complex. In addition, head movements are a crucial component in resolving ambiguous or confusing localization conditions (Thurlow et al., 1967; Wightman and Kistler, 1997; Begault, 1999).

The natural way for humans to hear the world is through active whole-body processes (Engel et al., 2013). Movement brings several improvements to auditory localization. Compared to static perception, a perceptual strategy that includes movement results in a richer and more varied percept. Although some early works reported equal or poorer sound localization during head movement (Wallach, 1940; Pollack and Rose, 1967; Simpson and Stanton, 1973), subsequent research has shown several benefits and has revealed the perceptual improvements permitted by perception during movement (Noble, 1981; Perrett and Noble, 1997a, b). Goosens and Van Opstal suggested that head movements could provide richer spatial information that allows listeners to update the internal representation of the sound and the environment (Goossens and Van Opstal, 1999). Some authors have also suggested that a perceptual advantage occurs only when the sound lasts long enough (Makous and Middlebrooks, 1990), with a minimum duration of the order of 2 s appearing to be necessary to allow subjects to achieve the conditions required for maximum performance (Thurlow and Mergener, 1970). Iwaya and colleagues also found that front-back confusion can only be effectively resolved with longer-lasting sounds (Iwaya et al., 2003). Some studies on acoustic localization have taken advantage of this condition for their experimental protocols: for example by using very short stimuli (typically ≤150 ms) to ensure that the sound ends before the subject can initiate a head movement, thus making it unnecessary to restrain the participant’s head (Carlile et al., 1997; Macpherson and Middlebrooks, 2000; Tabry et al., 2013; Oberem et al., 2020). Conversely, when the sound continues throughout the entire movement, the listener can implement a movement strategy within a closed-loop control paradigm (Otte et al., 2013).

Although both conditions of relative motion between source and listener bring about a perceptual advantage, there is a relative advantage in spatial processing when it is the listener who is moving (Brimijoin and Akeroyd, 2014). The presence of motion helps resolve or reduce ambiguities, such as front-back confusion (Wightman and Kistler, 1999; Begault et al., 2001; Iwaya et al., 2003; Brimijoin et al., 2010) and this is true even for listeners with cochlear implants (mainly through head movement) (Pastore et al., 2018). The relative motion improves the perception of distance (Loomis et al., 1990; Genzel et al., 2018), the perception of elevation (Perrett and Noble, 1997b), the effectiveness of HRTF systems (Loomis et al., 1990), and the assessment of one’s own movement (Speigle and Loomis, 1993).

## 4 Elements influencing auditory localization

### 4.1 Sound frequency spectrum

Acoustic localization performance is highly dependent on the frequency of the sound. Our perceptual system achieves the best localization accuracy for frequencies below 1,000 Hz and good localization accuracy for frequencies above 3,000 Hz. Localization accuracy decreases significantly in the range between 1,000 Hz and 3,000 Hz. These results are a consequence of the functional characteristics of our localization processes (see ITD and ILD). Experimental research such as that of Yost and Zhong, who tested frequencies of 250 Hz, 2,000 Hz, and 4,000 Hz, has confirmed the different localization abilities for the three frequency ranges (Yost and Zhong, 2014). ITD works best for frequencies below 1,500 Hz, while ILD is most effective for frequencies above 4,000 Hz.

Concerning the HRTF, Hebrank and Wright showed that sound information within the 4,000–16,000 Hz spectrum is necessary for good vertical localization. Langendijk and Bronkhorst consistently showed that the most important cues for vertical localization are in the 6,000–11000 Hz frequency range. More precisely, Blauert found that the presence of frequency components from about 8,000–10,000 Hz is critical for accurate estimation of elevation. Langendijk and Bronkhorst showed that antero-posterior localization cues occur in the 8,000–16,000 Hz range (Blauert, 1969; Hebrank and Wright, 1974; Langendijk and Bronkhorst, 2002).

The bandwidth of a sound plays an important role in acoustic localization: the broader the bandwidth, the better the localization performance (Coleman, 1968; Yost and Zhong, 2014) under both open-field and reverberant-room conditions (Hartmann, 1983). Furthermore, the spectral content of the sound is an important cue for estimating the distance of the sound source. This type of cue works under two different conditions. Over long distances, high frequencies are more attenuated than low frequencies due to propagation through the air. As a result, sounds with reduced high-frequency content are perceived as being farther away (Coleman, 1968; Butler et al., 1980; Little et al., 1992). However, in order to obtain a noticeable effect, the distance between the source and the listener must be greater than 15 m (Blauert, 1997). For sound sources close to the listener’s head (about 1 m), by contrast, the spectral content is modified due to the diffraction of the sound around the listener’s head. For this reason, for sources in the proximal space (<1.7 m), sounds at lower frequencies (<3,000 Hz) actually result in more accurate distance estimation than sounds at higher frequencies (>5,000 Hz) (Brungart and Rabinowitz, 1999; Kopčo and Shinn-Cunningham, 2011).

Finally, sound frequency appears to play a role in front-back confusion errors. Both Stevens and Newman, and Withington, found that the number of confusion errors was much higher for sound sources below 2,500 Hz. Letowski and Letowski reported more frequent errors for sound sources located near the sagittal plane for narrow-band sounds and for a spectral band below 8,000 Hz. The number of confusion errors decreases rapidly as the energy of the high-frequency component increases (Stevens and Newman, 1936; Withington, 1999; Letowski and Letowski, 2012).

### 4.2 Sound intensity

Sound intensity plays an important role in several aspects of auditory localization, and especially in determining the distance between the listener and the sound source. It does so by underpinning two important mechanisms: the estimation of the intensity of the direct wave, and the comparison between the intensities of the direct wave and the reverberated waves.

At the theoretical level, the intensity of a spherical wave falls by 6 dB with each doubling of distance (Warren, 1958; Warren et al., 1958). In the real word, however, both environmental factors and sound source features can alter this simple mathematical relationship (Zahorik et al., 2005). Experimental tests have shown that the reduction in intensity during propagation in air is greater than the theoretical value and that this reduction amounts to about 10 dB for each doubling of distance (Stevens and Guirao, 1962; Begault, 1991). However, Blauert found an even higher value of 20 dB (Blauert, 1997). Petersen confirmed that the relationship between intensity reduction and distance can be assumed to be linear (Petersen, 1990).

Some perceptual factors influence the accuracy with which we can estimate the distance to a sound source. The first, of course, is related to our ability to discriminate small changes in intensity. Research has shown that the smallest detectable change in intensity level for humans is about 0.4 dB for broadband noise, while this threshold increases to 1–2 dB for tonal sounds (this value varies with the frequency and sound level) (Riesz, 1932; Miller, 1947; Jesteadt et al., 1977).

It might be expected that a sound of higher intensity would always be easier to localize. However, research has shown that the ability to localize sounds in the median plane deteriorates above about 50 dB (Rakerd et al., 1998; Macpherson and Middlebrooks, 2000; Vliegen and Van Opstal, 2004). Performance degradation is more pronounced for short sounds and affects almost only the median plane – the reduction in localization accuracy on the left–right axis being much less pronounced. Some studies have found that at higher levels, localization performance improves again as the sound intensity increases. Marmel and colleagues tested localization ability at different sound intensity levels and compared artificial HRTF and free-field conditions. They found that in free-field listening, localization ability increases and then deteriorates monotonically up to 100 dB, whereas in the HRTF condition, performance still improves at 100 dB (Macpherson and Middlebrooks, 2000; Brungart and Simpson, 2008; Marmel et al., 2018).

The intensity value provides information relating to both the power and distance of the source. In the absence of information provided by other sensory channels, such as vision, this condition can lead to a state of indecision in the measurement of the two parameters. Researchers are still examining the way the auditory system handles the two pieces of information. The evidence produced by Zahorik and Wightman supports the hypothesis that the two processes are separate. These authors reported good power estimation even when distance estimation was less accurate (Zahorik and Wightman, 2001). The most commonly accepted way of resolving the confusion between power and distance is based on the Direct-to-Reverberant energy Ratio, which consists in a comparison between the direct wave and the reverberant wave train (see “Reverberation”) (Zahorik and Wightman, 2001).

### 4.3 Pointing methods

Research over the past 30 years has shown that pointing methods can affect precision and accuracy in localization tasks. Pointing paradigms can be classified as egocentric or allocentric, with egocentric methods generally being reported to be more accurate.

When defining a protocol for a localization task, several pointing/localizing methods are possible: the orientation of a body part, such as pointing with a hand or a finger (Pernaux et al., 2003; Finocchietti et al., 2015), the orientation of the chest (Haber et al., 1993), the nose (Middlebrooks, 1999b), or the head (Makous and Middlebrooks, 1990; Carlile et al., 1997; Begault et al., 2001); use of a hand-tool (Langendijk et al., 2001; Cappagli and Gori, 2016), or a computer interface (Pernaux et al., 2003; Schoeffler et al., 2014); walking (Loomis et al., 1998); or simply using a verbal response (Klatzky et al., 2003; Finocchietti et al., 2015). In 1955, Sandel and colleagues conducted three localization experiments in which participants gave the response using an acoustic pointer. The method made use of a mobile loudspeaker that participants could place at the location where they felt the stimulus had been emitted (Sandel et al., 1955).

Several studies have focused on evaluating or comparing different localization methods. In a study conducted on blind subjects, Haber and colleagues compared nine different response methods using pure tones as stimuli in the horizontal plane. They showed that using body parts as the pointing method provides the best performance by optimizing localization accuracy and reducing intersubject variability (Haber et al., 1993).

An interesting research was conducted by Lewald et al. (2000). They conducted some auditory localization experiments, investigating the influence of head rotation relative to the trunk. In the different experiments proposed, they used both headphones and an array of 9 speakers arranged in the azimuthal plane to deliver the sound stimuli. For the response, they tested head pointing, a laser pointer attached to the head, and a swivel pointer (which must be directed with both hands toward the sound source). The authors highlighted that sound localization is systematically underestimated (localization is biased toward the sagittal plane) when the head is oriented eccentrically. The orientation of the head on the azimuthal plane and the localization error appear almost linear. The presentation of virtual sources through headphones also showed similar deviations. When a visual reference of the head’s median plane was provided, sound localization was more accurate. Odegaard and colleagues used a very large sample of subjects (384 participants) in a study investigating the presence and direction of bias in both visual and auditory localizations. They used an eye tracking system to record participants’ responses. Contrary to Lewald, in the unimodal auditory condition they found a peripherally oriented localization bias (i.e., overestimation), which was also more pronounced as stimulus eccentricity increased (Odegaard et al., 2015). Recanzone and colleagues conducted comparative research, in which they found that the eccentricity of peripheral auditory targets is typically overestimated when using hand pointing, and typically underestimated when using head pointing methods. They suggested that the different relative position of the head with respect to the sound source and the trunk may explain these results (Recanzone et al., 1998).

One study show that the dominant hand also influences responses. The study by Ocklenburg investigated the effect of laterality in a sound localization task. The protocol is based on diffusion of the auditory stimuly through a set of 21 horizontal speakers, and a pointing by head orientation or hand pointing. Interestingly, results show that both right-and left-handers have a tendency to localize sound toward the side contralateral to the dominant hand, regardless of their overall accuracy (bias similar to that observed in visual perception, suggesting same supramodal neural processes involved) (Ocklenburg et al., 2010).

Majdak and colleagues used individualized HRTFs to compare head-and hand-pointing. In a virtual environment, they found that the pointing method had no significant effect on the localization task (Majdak et al., 2010). Tabry and colleagues also assessed head-and hand-pointing performance. They assessed the participants’ response to real words both in a free-field environment and in a semi-anechoic room. Under these conditions, and in contrast to Majdak’s findings, they found large and significant differences in performance between the two pointing methods. More specifically, they found better performance in the horizontal plane with the hand-pointing method, while head-pointing resulted in better performance in the vertical plane (Tabry et al., 2013). In addition, they reported lower accuracy for head-pointing at extreme upward and downward elevations, probably due to the greater difficulty of the articular movements.

Populin compared head-and gaze-pointing. He reported similar performances with the two methods in the most eccentric positions. However, in frontal positions, he unexpectedly found that gaze-pointing resulted in significantly larger errors than head-pointing (Populin, 2008).

Gilkey and colleagues proposed an original method using an allocentric paradigm called GELP (God’s Eyes Localization Pointing) designed to accelerate response collection in auditory-localization experiments. GELP uses a 20-cm-diameter sphere as a model of the listener’s head, on which the participant can indicate the direction from which he/she perceives the sound coming. Test results obtained with GELP showed that it was a fast way to record participants’ responses and that it was also more accurate than the verbal response method. However, when they compared their results with those of Makous and Middlebrooks (1990), the authors found that the GELP technique is significantly less accurate than the head-pointing technique (Makous and Middlebrooks, 1990; Gilkey et al., 1995). These results were subsequently confirmed by the work of Djelani et al. (2000).

To collect responses in a localization task, it is also possible to use a computer-controlled graphical interface (Graphical User Interface, GUI) through which participants can indicate the perceived direction. Pernaux and colleagues, and Schoeffler and colleagues, compared two different GUI methods, consisting of a 2D or 3D representation, with the participants using a mouse to give their responses. Both reported that the 3D version was more effective. Moreover, Pernaux also compared the finger-pointing method with the two previous methods and showed that finger-pointing was faster and more accurate (Pernaux et al., 2003; Schoeffler et al., 2014).

Table 1 shows and classifies a selection of articles that have investigated the characteristics of different pointing methods. This table provides an overview of the main categories into which the literature on auditory localization can be pragmatically classified. It also includes a selection of key reference works that illustrate these categories. The information catalogued in the table can serve as a framework for organizing new related work.

**Table 1:** Experimental research articles on pointing methods in auditory localization.

References	Pointing method					Spatial dimension	Auditory cue	Environ.
	Head (H), Gaze (G), Hand / Finger (HF), Hand Pointer Tool (T), Other (specified)							
	H	G	HF	T	Other	Azimuth (A) Elevation (E) Distance (D)		Real (R), Virtual (V)
Aggius-Vella et al. (2018)					Verbally	A		R
Aggius-Vella et al. (2020)					Verbally (f2)	A		R
Bahu et al. (2016)	×		×	× (a)		A, E		R
Begault et al. (2001)	×				Graph_Int (c)	A, E, D	HRTF	V
Berthomieu et al. (2019)					Verbally	D		V
Bidart and Lavandier (2016)					Scale (d), verbally	D		V
Boyer et al. (2013)	(×)		×			A		V
Brungart et al. (1999)					(e)	A, E	ITD, ILD, HRTF	V
Cappagli et al. (2017)					Verbally (f2)	D		R
Cappagli and Gori (2016)				×		A		R
Carlile et al. (1997)	×					A, E		R
Chandler and Grantham (1992)					PB, verbally (f2)	A, E, D		R
Djelani et al. (2000)	×		×		GELP	A, E	HRTF	V
Dobreva et al. (2011)					Joystick	A, E	ITD, ILD	R
Finocchietti et al. (2015)			×			A, E		R
Getzmann (2003)			×		Verbally	E		R
Gilkey et al. (1995)					GELP	A, E		R
Guo et al. (2019)					Verbally	(A), D		R
Han and Chen (2019)					Verbally (f2)	A	HRTF	V
Klingel et al. (2021)					Keyboard (f2)	A	ITD, ILD	V
Langendijk et al. (2001)				× (a)		A, E		V
Lewald et al. (2000)	×			×	Laser pointer	A		R,V
Loomis et al. (1998)					Verbally, walk (g)	D		R
Macpherson (1994)					Verbally	(A), E	HRTF	R
Macpherson and Middlebrooks (2000)	×					(A), E		R
Majdak et al. (2010)	×			×		A, E, D	HRTF	V
Makous and Middlebrooks (1990)	×					A, E		R
Mershon and King (1975)					Writing	D		R
Middlebrooks (1999b)	×				(i)	A, E	HRTF	R,V
Nilsson and Schenkman (2016)					Keyboard (f2)	A	ITD, ILD	V
Oberem et al. (2020)	×			× (b)		E	HRTF	V
Ocklenburg et al. (2010)	×			×		A		R
Odegaard et al. (2015)		×				A		R
Otte et al. (2013)	×					A, E		R
Pernaux et al. (2003)			×		Graph_Int (c)	A, E		V
Populin (2008)		×				A, E		R
Rajendran and Gamper (2019)					Keyboard (f3)	E	HRTF	V
Recanzone et al. (1998)	×				Switch	A		R
Rhodes (1987)					Verbally	A		R
Risoud et al. (2020)					Verbally	A	ITD, ILD, HRTF	R
Rummukainen et al. (2018)					Verbally (f2)	A	ITD, ILD	V
Schoeffler et al. (2014)					Graph_Int (c)	A, E		R
Spiousas et al. (2017)					Verbally	D		R
Tabry et al. (2013)	×			×		A, E		R
Yost (2016)					Keyboard (f4)	A		R
Zahorik (2002)					Writing (h)	D		R
Van Wanrooij and Van Opstal (2004)	×					A, E	HRTF	R

The table presents a selection of research articles on experimental auditory localization and the pointing methods used. The selected articles propose a comparison of different pointing methods or provide information about a specific method. The Pointing method column provides the used or analyzed pointing methods [Head (H), Gaze (G), Hand/Finger (F), Hand Pointer Tool (T), Other (specified)]. The Spatial dimension column indicates which spatial dimensions are considered [azimuth (A), elevation (E), distance (D)]. The parentheses indicate that the variable is a part of the assessment, even though it is not the main object of the research. The Auditory cue column indicates whether the focus of the article is on the analysis of a specific auditory cue: ITD, ILD, HRTF. The Environ. column indicates whether the test was conducted in a real environment (R), using loudspeakers placed in real space around the listener, or in a virtual environment (V), with the listener wearing audio headphones and using HRTF techniques. “Graph_Int” for Graphical Interface. “PB” for Push Button. (a) Gun pointer. (b) Hand-held marker. (c) Schematic 2D and 3D views on which the subject reported his localization judgment with a mouse or a joystick. (d) Representative scale with hand selection. (e) HRTF measurement. (f2) Two-alternative forced choice. (f3) Three-alternative forced choice. (f4) Fifteen-alternative forces choice. (g) Listener had to walk to the location perceived as the source. (h) On a computer terminal with a numeric keypad. (i) Listener was instructed to “point with her/his nose.”

### 4.4 Training

The considerable research and extensive experimental tests conducted in recent decades have shown that habituation and training are factors that significantly influence participants’ performance in localization tasks. Habituation allows participants to become familiar with the task and the materials used, and a few trials are usually enough. Training is a deeper process that aims to enable participants to “appropriate” the methods and stimuli and requires a much greater number of trials (Kacelnik et al., 2006; Kumpik et al., 2010). There is no consensus on the duration required for an effective initial training phase. The training task plays an equally important role. For instance, Hüg et al. (2022) investigated the effect of training methods on auditory localization performance for the distance dimension. Comparing active and passive movements, they observed that the training was effective in improving localization performance only with the active method (Hüg et al., 2022). Deep and effective training results in much lower variability in the results. However, from an ecological point of view, deep training may affect the spontaneity of responses (Neuhoff, 2004).

Although some authors prefer not to subject their participants to a training phase, thus prioritizing unconditioned responses, this approach appears to be very uncommon (Mershon and King, 1975; Populin, 2008). Some studies have foregone the use of a habituation or training phase and have instead performed a calibration and/or verification of task understanding (Otte et al., 2013). Bahu et al. and Hüg et al. proposed a simple habituation phase consisting of 10 or 4 trials, respectively, that were identical to the task used in the subsequent test (Bahu et al., 2016; Hüg et al., 2022). Macpherson and Middlebrooks proposed training consisting of five consecutive phases, each composed of 60 trials. The five phases progressively introduced the participant to the complete task. The entire training phase lasted 10 min and was performed immediately before the tests (Macpherson and Middlebrooks, 2000).

Other studies, by contrast, have proposed a more extensive training phase. In a study specifically devoted to the effects of training on auditory localization abilities, Majdak and colleagues, found that for the head-and hand-pointing methods, respectively, listeners needed 590 and 710 trials (on average) to achieve the required performance (Majdak et al., 2010). To enable their participants to learn how to use the pointing method correctly, Oberem and colleagues proposed training consisting of 600 localization trials with feedback (Oberem et al., 2020). Middlebrooks trained participants with 1,200 trials (Middlebrooks, 1999b). Oldfield and Parker’s participants were trained for at least 2 h before performing the test (Oldfield and Parker, 1984). Makous and Middlebrooks administered 10 to 20 training sessions to listeners, with and without feedback (Makous and Middlebrooks, 1990).

### 4.5 Auditory localization illusions

In auditory illusions, the perception or interpretation of a sound is not consistent with the actual sound in terms of its physical, spatial, or other characteristics. Some auditory illusions concern the localization or lateralization of sound. One of the earliest and best-documented auditory illusions is the Octave illusion (or Deutsch illusion), discovered by Diana Deutsch in 1973. Deutsch has identified a large number of auditory illusions of different types, of which the Octave illusion is the best known. This illusion is produced by playing a “high” and a “low” tone through stereo headphones, while alternating the sound-ear correspondence (“high” left and “low” right, and *vice-versa*) four times per second. The two tones are an octave apart. The illusion takes the form of a perceptual alteration of the nature and lateralization of the sounds, which are perceived as a single tone that continuously alternates between the right and left ears (Deutsch, 1974, 2004). Although the explanation of this illusion is still a matter of debate, the most widely accepted solution is the one proposed by the author herself and derives from the existence of a conflict between the “what” and “where” decision-making mechanisms (Deutsch, 1975). One of the most robust and fascinating auditory illusions is the Franssen effect, discovered by Nico Valentinus Franssen in 1960. The Franssen effect is created by playing a sound through two loudspeakers, resulting in an auditory illusion in which the listener mislocalizes the lateralization of the sound. At the beginning of the illusion, a sound is emitted from only one of the loudspeakers (it is unimportant whether this is the left or right speaker) before then being completely transferred to the opposite side. Although the first speaker has stopped playing, the listener does not perceive the change of side. The most widely accepted explanation of the Franssen effect identifies the use of a pure sound, the change in laterality through “rapid fading” from one side to the other, the dominance of onsets for localization (in accordance with the law of the first wave front) and, most importantly, the presence of reverberation as the key elements. In the absence of reverberation, the effect does not occur (Hartmann and Rakerd, 1989b). The illusion created by the Franssen effect is an excellent example of how perception (and in this particular case, auditory localization) also arises from the individual’s prior experience and is not just the result of momentary stimulation.

Advances in the understanding of the functioning of the auditory system have stimulated new and more original research, and this has led to the discovery (or creation) of new auditory illusions. Bloom studied and experimented with the perception of elevation; he created an illusion of sound elevation through spectral manipulation of the sound (Bloom, 1977). A more recent auditory illusion is known as the Transverse-and-bounce illusion. This illusion uses front-to-back confusion and volume changes to create the perception that a single sound stimulus is in motion. When the volume increases, the sound is perceived as approaching, while when it decreases, it is perceived as moving away from the listener. This illusion can be reproduced using either speakers or headphones (Bainbridge et al., 2015). Di Zio and colleagues investigated the Audiogravic Illusion (i.e., head-centered auditory localization influenced by the intensity and direction of gravity). To do this, they used an original and interesting experimental setup to manipulate the direction of gravity perceived by participants. The results of their research show that by increasing the magnitude of the resulting gravitational force and changing its direction relative to the head and torso, it is possible to obtain an apparent displacement of a sound relative to the head in the opposite direction (DiZio et al., 2001).

Some auditory illusions have subsequently been used in a number of important applications. Stereophony is perhaps the most widely used illusion. Stereophony is based on the “summing localization” effect: when two sounds reach the two ears with a ‘limited incoherence’ in time and level, the stimuli are merged into a single percept. Under these conditions, our brain infers a “phantom source,” located away from the listener, whose location is consistent with the perceived differences between the right and left ear stimuli. The purpose of using this illusion is to achieve a wider spatial perception in the diffusion of sounds and music with headphones or speakers (Chernyak and Dubrovsky, 1968).

## 5 Conclusion

The human ability to localize sounds in our surroundings is a complex and fascinating phenomenon. Through a sophisticated set of mechanisms, our auditory system enables us to perceive the spatial location of sounds and orient ourselves in the world around us.

In this article, we examined the main processes involved in auditory localization, based on monoaural and binaural cues, time and intensity differences between the ears, and frequencies that make it easier – or more difficult – to localize the source. We have supplemented the “traditional” description of these mechanisms with the most recent research findings, which show how some ancillary cues, such as reverberation or relative motion, are essential to achieve our impressive localization performance. We also have enhanced the functional description with relevant information concerning methodologies and perceptual limitations in order to provide a broader information set.

Modern applications of this knowledge make it possible today to live remarkable experiences. In particular, HRTF promises excellent spatialization results, but requires better understanding and management of its artificial reproduction. Resolving some conditions of localization uncertainty, and easily customizing equations on each listener, are still open challenges.

In the present and in the future, one of the most interesting ethical applications concerns the perceptual support for people with disabilities. Providing more effective assistive devices is certainly one of the most exciting challenges, as in the case of auditory rehabilitation and assistive devices, such as sensory substitution devices for the blind (Bordeau et al., 2023).

## Author contributions

AC: Conceptualization, Data curation, Methodology, Project administration, Supervision, Writing – original draft, Writing – review & editing. CB: Data curation, Formal analysis, Investigation, Writing – original draft, Writing – review & editing. MA: Conceptualization, Data curation, Funding acquisition, Resources, Supervision, Writing – original draft, Writing – review & editing.

## References

[ref1] Acosta A. Grijalva F. Alvarez R. Acuna B. (2020). Bilinear and triangular spherical head-related transfer functions interpolation on non-uniform meshes. 2020 IEEE ANDESCON, ANDESCON, Quito, Ecuador.

[ref2] Aggius-Vella E. Campus C. Gori M. (2018). Different audio spatial metric representation around the body. Sci. Rep. 8, 1–9. doi: 10.1038/s41598-018-27370-9 29925849 PMC6010478

[ref3] Aggius-Vella E. Gori M. Campus C. Moore B. C. J. Pardhan S. Kolarik A. J.. (2022). Auditory distance perception in front and rear space. Hear. Res. 417:108468. doi: 10.1016/j.heares.2022.108468, PMID: 35220107

[ref4] Aggius-Vella E. Kolarik A. J. Gori M. Cirstea S. Campus C. Moore B. C. J.. (2020). Comparison of auditory spatial bisection and minimum audible angle in front, lateral, and back space. Sci. Rep. 10:6279. doi: 10.1038/s41598-020-62983-z, PMID: 32286362 PMC7156409

[ref5] Ahveninen J. Kopčo N. Jääskeläinen I. P. (2014). Psychophysics and neuronal bases of sound localization in humans. Hear. Res. 307, 86–97. doi: 10.1016/j.heares.2013.07.008, PMID: 23886698 PMC3858499

[ref6] Andersen J. S. Miccini R. Serafin S. Spagnol S. (2021). Evaluation of individualized HRTFs in a 3D shooter game. In 2021 immersive and 3D audio: from architecture to automotive, I3DA, IEEE. 2021.

[ref7] Bahu H. Carpentier T. Noisternig M. Warusfel O. (2016). Comparison of different egocentric pointing methods for 3D sound localization experiments. Acta Acust. 102, 107–118. doi: 10.3813/AAA.918928

[ref8] Bainbridge C. M. Bainbridge W. A. Oliva A. (2015). Quadri-stability of a spatially ambiguous auditory illusion. Front. Hum. Neurosci. 8:1060. doi: 10.3389/fnhum.2014.01060, PMID: 25642180 PMC4295545

[ref9] Begault D. R. (1991). Preferred sound intensity increase for sensation of half distance. Percept. Mot. Skills 72, 1019–1029. doi: 10.2466/pms.1991.72.3.1019, PMID: 1891303

[ref10] Begault D. R. (1992). Perceptual effects of synthetic reverberation on three-dimensional audio systems. AES J. Audio Eng. Soc. 40, 895–904.

[ref11] Begault D. R. (1999). Auditory and non-auditory factors that potentially influence virtual acoustic imagery. AES 16th Int. Conf, Moffett Field, CA.

[ref12] Begault D. R. (2000). 3-D sound for virtual reality and multimedia. Moffett Field, CA: Ames Research Center.

[ref13] Begault D. R. Wenzel E. M. Anderson M. R. (2001). Direct comparison of the impact of head tracking, reverberation, and individualized head-related transfer functions on the spatial perception of a virtual speech source. J. Audio Eng. Soc. 49, 904–916. 11885605

[ref14] Berger C. C. Gonzalez-Franco M. Tajadura-Jiménez A. Florencio D. Zhang Z. (2018). Generic HRTFs may be good enough in virtual reality. Improving source localization through cross-modal plasticity. Front. Neurosci. 12:21. doi: 10.3389/fnins.2018.00021 29456486 PMC5801410

[ref15] Bernstein L. R. Trahiotis C. (1985). Lateralization of low-frequency, complex waveforms: the use of envelope-based temporal disparities. J. Acoust. Soc. Am. 77, 1868–1880. doi: 10.1121/1.391938, PMID: 3998297

[ref16] Best V. Baumgartner R. Lavandier M. Majdak P. Kopčo N. (2020). Sound externalization: a review of recent research. Trends Hear. 24:233121652094839. doi: 10.1177/2331216520948390, PMID: 32914708 PMC7488874

[ref9001] Berthomieu G. Koehl V. Paquier M. (2019). Loudness and distance estimates for noise bursts coming from several distances with and without visual cues to their source. Universitätsbibliothek der RWTH Aachen.

[ref9002] Bidart A. Lavandier M. (2016). Room-induced cues for the perception of virtual auditory distance with stimuli equalized in level. Acta Acustica United with Acustica, 102, 159–169.

[ref17] Blauert J. (1969). Sound localization in the median plane. Acust. 22, 205–213.

[ref18] Blauert J. (1996). The psychophysics of human sound localization. Spat. Heraing, Revis. Ed. Spatial hearing. MIT press.

[ref19] Blauert J. (1997). Spatial hearing: the psychophysics of human sound localization.

[ref20] Bloom P. J. (1977). Creating source elevation illusions by spectral manipulation. J. Audio Eng. Soc. 25, 560–565.

[ref21] Bordeau C. Scalvini F. Migniot C. Dubois J. Ambard M. (2023). Cross-modal correspondence enhances elevation localization in visual-to-auditory sensory substitution. Front. Psychol. 14:1079998. doi: 10.3389/fpsyg.2023.1079998, PMID: 36777233 PMC9909421

[ref22] Boyer E. O. Babayan B. M. Bevilacqua F. Noisternig M. Warusfel O. Roby-Brami A.. (2013). From ear to hand: the role of the auditory-motor loop in pointing to an auditory source. Front. Comput. Neurosci. 7:26. doi: 10.3389/fncom.2013.00026, PMID: 23626532 PMC3631711

[ref23] Brimijoin W. O. Akeroyd M. A. (2014). The moving minimum audible angle is smaller during self motion than during source motion. Front. Neurosci. 8:273. doi: 10.3389/fnins.2014.00273, PMID: 25228856 PMC4151253

[ref24] Brimijoin W. O. McShefferty D. Akeroyd M. A. (2010). Auditory and visual orienting responses in listeners with and without hearing-impairment. J. Acoust. Soc. Am. 127, 3678–3688. doi: 10.1121/1.3409488, PMID: 20550266 PMC4338612

[ref25] Bronkhorst A. W. (1995). Localization of real and virtual sound sources. J. Acoust. Soc. Am. 98, 2542–2553. doi: 10.1121/1.413219

[ref26] Bronkhorst A. W. Houtgast T. (1999). Auditory distance perception in rooms. Nature 397, 517–520. doi: 10.1038/17374, PMID: 10028966

[ref27] Brughera A. Dunai L. Hartmann W. M. (2013). Human interaural time difference thresholds for sine tones: the high-frequency limit. J. Acoust. Soc. Am. 133, 2839–2855. doi: 10.1121/1.4795778, PMID: 23654390 PMC3663869

[ref28] Brungart D. S. Durlach N. I. Rabinowitz W. M. (1999). Auditory localization of nearby sources. II. Localization of a broadband source. J. Acoust. Soc. Am. 106, 1956–1968. doi: 10.1121/1.427943 10530020

[ref29] Brungart D. S. Rabinowitz W. M. (1999). Auditory localization of nearby sources. Head-related transfer functions. J. Acoust. Soc. Am. 106, 1465–1479. doi: 10.1121/1.427180 10489704

[ref30] Brungart D. S. Simpson B. D. (2008). Effects of temporal fine structure on the localization of broadband sounds: potential implications for the Design of Spatial Audio Displays. Proceedings of the 14th International Conference on Auditory Display, Paris, France June 24–27, 2008. Available at: https://www.icad.org/Proceedings/2008/BrungartSimpson2008b.pdf

[ref31] Brungart D. S. Simpson B. D. Kordik A. J. (2005). Localization in the presence of multiple simultaneous sounds. Acta Acust. 91, 471–479.

[ref32] Bruns P. Thun C. Röder B. (2024). Quantifying accuracy and precision from continuous response data in studies of spatial perception and crossmodal recalibration. Behav. Res. Methods 56, 3814–3830. doi: 10.3758/s13428-024-02416-1 38684625 PMC11133116

[ref33] Butler R. A. (1986). The bandwidth effect on monaural and binaural localization. Hear. Res. 21, 67–73. doi: 10.1016/0378-5955(86)90047-X, PMID: 3957797

[ref34] Butler R. A. Levy E. T. Neff W. D. (1980). Apparent distance of sounds recorded in echoic and anechoic chambers. J. Exp. Psychol. Hum. Percept. Perform. 6, 745–750. doi: 10.1037/0096-1523.6.4.745, PMID: 6449541

[ref35] Cappagli G. Gori M. (2016). Auditory spatial localization: developmental delay in children with visual impairments. Res. Dev. Disabil. 53-54, 391–398. doi: 10.1016/j.ridd.2016.02.019, PMID: 27002960

[ref9003] Cappagli G. Cocchi E. Gori M. (2017). Auditory and proprioceptive spatial impairments in blind children and adults. Dev Sci, 20: e12374. 10.1111/desc.12374 26613827

[ref36] Carlile S. Leong P. Hyams S. (1997). The nature and distribution of errors in sound localization by human listeners. Hear. Res. 114, 179–196. doi: 10.1016/S0378-5955(97)00161-5, PMID: 9447931

[ref37] Catic J. Santurette S. Buchholz J. M. Gran F. Dau T. (2013). The effect of interaural-level-difference fluctuations on the externalization of sound. J. Acoust. Soc. Am. 134, 1232–1241. doi: 10.1121/1.4812264, PMID: 23927121

[ref38] Catic J. Santurette S. Dau T. (2015). The role of reverberation-related binaural cues in the externalization of speech. J. Acoust. Soc. Am. 138, 1154–1167. doi: 10.1121/1.4928132, PMID: 26328729

[ref9004] Chandler D. W. Grantham D. W. (1992). Minimum audible movement angle in the horizontal plane as a function of stimulus frequency and bandwidth, source azimuth, and velocity. J. Acoust. Soc. Am, 91, 1624–1636. 1564199 10.1121/1.402443

[ref39] Chernyak R. I. Dubrovsky N. A.. (1968). Pattern of the noise images and the binaural summation of loudness for the different interaural correlation of noise. Proceedings of the 6th International Congress on Acoustics, Tokyo.

[ref40] Chun C. J. Moon J. M. Lee G. W. Kim N. K. Kim H. K. (2017). Deep neural network based HRTF personalization using anthropometric measurements. In 143rd audio engineering society convention 2017, AES, 2017.

[ref41] Coleman P. D. (1968). Dual Rôle of frequency Spectrum in determination of auditory distance. J. Acoust. Soc. Am. 44, 631–632. doi: 10.1121/1.1911132, PMID: 5665535

[ref42] Demirkaplan Ö. Haclhabibogˇlu H. (2020). Effects of interpersonal familiarity on the auditory distance perception of level-equalized reverberant speech. Acta Acust. 4:26. doi: 10.1051/aacus/2020025

[ref43] Deutsch D. (1974). An auditory illusion. Nature 251, 307–309. doi: 10.1038/251307a0, PMID: 4427654

[ref44] Deutsch D. (1975). Musical Illusions. Sci. Am. 233, 92–104. doi: 10.1038/scientificamerican1075-92, PMID: 1162325

[ref45] Deutsch D. (2004). The octave illusion revisited again. J. Exp. Psychol. Hum. Percept. Perform. 30, 355–364. doi: 10.1037/0096-1523.30.2.355, PMID: 15053694

[ref46] DiZio P. Held R. Lackner J. R. Shinn-Cunningham B. Durlach N. (2001). Gravitoinertial force magnitude and direction influence head-centric auditory localization. J. Neurophysiol. 85, 2455–2460. doi: 10.1152/jn.2001.85.6.2455, PMID: 11387391

[ref47] Djelani T. Pörschmann C. Sahrhage J. Blauert J. (2000). An interactive virtual-environment generator for psychoacoustic research II: Collection of head-related impulse responses and evaluation of auditory localization: Acustica. 86, 1046–1053.

[ref48] Dobreva M. S. O’Neill W. E. Paige G. D. (2011). Influence of aging on human sound localization. J. Neurophysiol. 105, 2471–2486. doi: 10.1152/jn.00951.2010, PMID: 21368004 PMC3094163

[ref49] Durlach N. I. Rigopulos A. Pang X. D. Woods W. S. Kulkarni A. Colburn H. S.. (1992). On the externalization of auditory images. Presence 1, 251–257. doi: 10.1162/pres.1992.1.2.251, PMID: 36471207

[ref50] Elpern B. S. Naunton R. F. (1964). Lateralizing effects of Interaural phase differences. J. Acoust. Soc. Am. 36, 1392–1393. doi: 10.1121/1.1919215

[ref51] Engel A. K. Maye A. Kurthen M. König P. (2013). Where’s the action? The pragmatic turn in cognitive science. Trends Cogn. Sci. 17, 202–209. doi: 10.1016/j.tics.2013.03.006, PMID: 23608361

[ref52] Febretti A. Nishimoto A. Thigpen T. Talandis J. Long L. Pirtle J. D.. (2013). CAVE2: A hybrid reality environment for immersive simulation and information analysis. in The Engineering Reality of Virtual Reality 2013SPIE. 8649, 9–20.

[ref53] Finocchietti S. Cappagli G. Gori M. (2015). Encoding audio motion: spatial impairment in early blind individuals. Front. Psychol. 6:1357. doi: 10.3389/fpsyg.2015.01357, PMID: 26441733 PMC4561343

[ref54] Fitzgibbons P. J. Gordon-Salant S. (2010). Behavioral studies with aging humans: hearing sensitivity and psychoacoustics. The aging auditory system, 111–134.

[ref55] Fletcher H. Munson W. A. (1933). Loudness, its definition, measurement and calculation. J. Acoust. Soc. Am. 5, 82–108. doi: 10.1121/1.1915637

[ref56] Fontana F. Rocchesso D. (2008). Auditory distance perception in an acoustic pipe. ACM Trans. Appl. Percept. 5, 1–15. doi: 10.1145/1402236.1402240

[ref57] Freeland F. Wagner L. Diniz P. (2002). Efficient HRTF interpolation in 3D moving sound. 22nd Int. Conf. Virtual, Synth. Entertain. Audio Audio Eng. Soc.

[ref58] Galati G. Pelle G. Berthoz A. Committeri G. (2010). Multiple reference frames used by the human brain for spatial perception and memory. Exp. Brain Res. 206, 109–120. doi: 10.1007/s00221-010-2168-8, PMID: 20186405

[ref59] Gan W.-S. Peksi S. He J. Ranjan R. Duy Hai N. Kumar Chaudhary N. (2017). Personalized HRTF measurement and 3D audio rendering for AR/VR headsets. In Audio Engineering Society Convention 142. Audio Engineering Society.

[ref60] Garcia S. E. Jones P. R. Rubin G. S. Nardini M. (2017). Auditory localisation biases increase with sensory uncertainty. Sci. Rep. 7, 1–10. doi: 10.1038/srep40567, PMID: 28074913 PMC5225420

[ref61] Gardner W. G. (1995). Efficient convolution without input-output delay. J. Audio Eng. Soc., 43, 127–136.

[ref62] Gelfand S. A. (2017). Hearing: an introduction to psychological and physiological acoustics. CRC Press.

[ref63] Genzel D. Schutte M. Brimijoin W. O. MacNeilage P. R. Wiegrebe L. (2018). Psychophysical evidence for auditory motion parallax. Proc. Natl. Acad. Sci. U. S. A. 115, 4264–4269. doi: 10.1073/pnas.1712058115, PMID: 29531082 PMC5910811

[ref64] Geronazzo M. Sikstrom E. Kleimola J. Avanzini F. De Gotzen A. Serafin S. (2019). The impact of an accurate vertical localization with HRTFs on short explorations of immersive virtual reality scenarios. In proceedings of the 2018 IEEE international symposium on mixed and augmented reality, ISMAR, IEEE, 2018 90–97.

[ref9005] Getzmann S. (2003). The influence of the acoustic context on vertical sound localization in the median plane. Percept. psychophys, 65, 1045–1057. 14674632 10.3758/bf03194833

[ref65] Giguere C. Abel S. M. (1993). Sound localization: effects of reverberation time, speaker array, stimulus frequency, and stimulus rise/decay. J. Acoust. Soc. Am. 94, 769–776. doi: 10.1121/1.408206, PMID: 8370883

[ref66] Gilkey R. H. Good M. D. Ericson M. A. Brinkman J. Stewart J. M. (1995). A pointing technique for rapidly collecting localization responses in auditory research. Behav. Res. Methods Instrum. Comput. 27, 1–11. doi: 10.3758/BF03203614

[ref67] Glasberg B. R. Moore B. C. J. (1990). Derivation of auditory filter shapes from notched-noise data. Hear. Res. 47, 103–138. doi: 10.1016/0378-5955(90)90170-T, PMID: 2228789

[ref68] Goossens H. H. L. M. Van Opstal A. J. (1999). Influence of head position on the spatial representation of acoustic targets. J. Neurophysiol. 81, 2720–2736. doi: 10.1152/jn.1999.81.6.2720, PMID: 10368392

[ref69] Graziano M. S. A. (2001). Is reaching eye-centered, body-centered, hand-centered, or a combination? Rev. Neurosci. 12, 175–185. doi: 10.1515/REVNEURO.2001.12.2.175 11392457

[ref70] Grijalva F. Martini L. C. Florencio D. Goldenstein S. (2017). Interpolation of head-related transfer functions using manifold learning. IEEE Signal Process. Lett. 24, 221–225. doi: 10.1109/LSP.2017.2648794

[ref71] Grothe B. Pecka M. (2014). The natural history of sound localization in mammals-a story of neuronal inhibition. Front. Neural Circ. 8:116. doi: 10.3389/fncir.2014.00116, PMID: 25324726 PMC4181121

[ref72] Grothe B. Pecka M. McAlpine D. (2010). Mechanisms of sound localization in mammals. Physiol. Rev. 90, 983–1012. doi: 10.1152/physrev.00026.2009, PMID: 20664077

[ref73] Guo Z. Lu Y. Wang L. Yu G. (2019). Discrimination experiment of sound distance perception for a real source in near-field, In EAA Spatial Audio Signal Processing Symposium (pp. 85–89).

[ref74] Guski R. (1990). Auditory localization: effects of reflecting surfaces. Perception 19, 819–830. doi: 10.1068/p190819, PMID: 2130378

[ref75] Haber L. Haber R. N. Penningroth S. Novak K. Radgowski H. (1993). Comparison of nine methods of indicating the direction to objects: data from blind adults. Perception 22, 35–47. doi: 10.1068/p220035, PMID: 8474833

[ref9006] Han Y. Chen F. (2019). Minimum audible movement angle in virtual auditory environment: Effect of stimulus frequency. In 2019 IEEE Conference on Multimedia Information Processing and Retrieval (MIPR), IEEE. 175–178.

[ref76] Hartmann W. M. (1983). Localization of sound in rooms. J. Acoust. Soc. Am. 74, 1380–1391. doi: 10.1121/1.390163 6643850

[ref77] Hartmann W. M. (1999). How we localize sound. Phys. Today 52, 24–29. doi: 10.1063/1.882727, PMID: 38879756

[ref78] Hartmann W. M. Macaulay E. J. (2014). Anatomical limits on interaural time differences: an ecological perspective. Front. Neurosci. 8:34. doi: 10.3389/fnins.2014.00034, PMID: 24592209 PMC3937989

[ref79] Hartmann W. M. Rakerd B. (1989a). On the minimum audible angle—a decision theory approach. J. Acoust. Soc. Am. 85, 2031–2041. doi: 10.1121/1.397855, PMID: 2732384

[ref80] Hartmann W. M. Rakerd B. (1989b). Localization of sound in rooms IV: the Franssen effect. J. Acoust. Soc. Am. 86, 1366–1373. doi: 10.1121/1.398696, PMID: 2808910

[ref81] Hartmann W. M. Rakerd B. Crawford Z. D. Zhang P. X. (2016). Transaural experiments and a revised duplex theory for the localization of low-frequency tones. J. Acoust. Soc. Am. 139, 968–985. doi: 10.1121/1.4941915, PMID: 26936576 PMC4769260

[ref82] Hartmann W. M. Rakerd B. Macaulay E. J. (2013). On the ecological interpretation of limits of interaural time difference sensitivity. in Proceedings of Meetings on Acoustics. (Vol. 19, No. 1). AIP Publishing.

[ref83] Hebrank J. Wright D. (1974). Spectral cues used in the localization of sound sources on the median plane. J. Acoust. Soc. Am. 56, 1829–1834. doi: 10.1121/1.1903520, PMID: 4443482

[ref84] Heinz M. G. Colburn H. S. Carney L. H. (2001). Evaluating auditory performance limits: i. one-parameter discrimination using a computational model for the auditory nerve. Neural Comput. 13, 2273–2316. doi: 10.1162/089976601750541804 11570999

[ref85] Howard D. M. Angus J. A. S. (2017). Acoustics and psychoacoustics. 5th Edn. Routledge.

[ref86] Howarth A. Shone G. R. (2006). Ageing and the auditory system. Postgrad. Med. J. 82, 166–171. doi: 10.1136/pgmj.2005.039388, PMID: 16517797 PMC2563709

[ref87] Hüg M. X. Bermejo F. Tommasini F. C. Di Paolo E. A. (2022). Effects of guided exploration on reaching measures of auditory peripersonal space. Front. Psychol. 13:983189. doi: 10.3389/fpsyg.2022.983189, PMID: 36337523 PMC9632294

[ref88] Iwaya Y. Suzuki Y. Kimura D. (2003). Effects of head movement on front-back error in sound localization. Acoust. Sci. Technol. 24, 322–324. doi: 10.1250/ast.24.322, PMID: 36816108

[ref89] Jerath R. Crawford M. W. Barnes V. A. (2015). Functional representation of vision within the mind: a visual consciousness model based in 3D default space. Majallah-i Īrānī-i nazẓarīyah pardāzī dar ʻulūm-i pizishkī 9, 45–56. doi: 10.1016/j.jmhi.2015.02.001, PMID: 27959269

[ref90] Jesteadt W. Wier C. C. Green D. M. (1977). Intensity discrimination as a function of frequency and sensation level. J. Acoust. Soc. Am. 61, 169–177. doi: 10.1121/1.381278 833368

[ref91] Kacelnik O. Nodal F. R. Parsons C. H. King A. J. (2006). Training-induced plasticity of auditory localization in adult mammals. PLoS Biol. 4:e71. doi: 10.1371/journal.pbio.0040071, PMID: 16509769 PMC1393755

[ref92] Kates J. M. Arehart K. H. (2018). Improving auditory externalization for hearing-aid remote microphones. In conference record of 51st Asilomar conference on signals, systems and computers, ACSSC, IEEE. 2017.

[ref93] Kearney G. Gorzel M. Rice H. Boland F. (2012). Distance perception in interactive virtual acoustic environments using first and higher order ambisonic sound fields. Acta Acust United Acust 98, 61–71. doi: 10.3813/AAA.918492

[ref94] Klatzky R. L. Lippa Y. Loomis J. M. Golledge R. G. (2003). Encoding, learning, and spatial updating of multiple object locations specified by 3-D sound, spatial language, and vision. Exp. Brain Res. 149, 48–61. doi: 10.1007/s00221-002-1334-z, PMID: 12592503

[ref9007] Klingel M. Kopčo N. Laback B. (2021). Reweighting of binaural localization cues induced by lateralization training. J Assoc Res Otolaryngol, 22, 551–566. 33959826 10.1007/s10162-021-00800-8 PMC8476684

[ref95] Klumpp R. G. Eady H. R. (1956). Some measurements of Interaural time difference thresholds. J. Acoust. Soc. Am. 28, 859–860. doi: 10.1121/1.1908493

[ref96] Kopčo N. Shinn-Cunningham B. G. (2011). Effect of stimulus spectrum on distance perception for nearby sources. J. Acoust. Soc. Am. 130, 1530–1541. doi: 10.1121/1.3613705, PMID: 21895092 PMC3188969

[ref97] Kumpik D. P. Kacelnik O. King A. J. (2010). Adaptive reweighting of auditory localization cues in response to chronic unilateral earplugging in humans. J. Neurosci. 30, 4883–4894. doi: 10.1523/JNEUROSCI.5488-09.2010, PMID: 20371808 PMC4225134

[ref98] Laird D. A. Taylor E. Wille H. H. (1932). The apparent reduction of loudness. J. Acoust. Soc. Am. 3, 393–401. doi: 10.1121/1.1915570

[ref99] Langendijk E. H. A. Bronkhorst A. W. (2002). Contribution of spectral cues to human sound localization. J. Acoust. Soc. Am. 112, 1583–1596. doi: 10.1121/1.1501901 12398464

[ref100] Langendijk E. H. A. Kistler D. J. Wightman F. L. (2001). Sound localization in the presence of one or two distracters. J. Acoust. Soc. Am. 109, 2123–2134. doi: 10.1121/1.1356025 11386564

[ref101] Lee G. W. Kim H. K. (2018). Personalized HRTF modeling based on deep neural network using anthropometric measurements and images of the ear. Appl. Sci. 8:2180. doi: 10.3390/app8112180

[ref102] Letowski T. Letowski S. (2011). “Localization error: accuracy and precision of auditory localization” in Advances in sound localization. 55, 55–78.

[ref103] Letowski T. R. Letowski S. T. (2012). Auditory spatial perception: auditory localization. Army Research Laboratory Aberdeen Proving Ground MD Human Research and Engineering Directorate.

[ref104] Lewald J. Dörrscheidt G. J. Ehrenstein W. H. (2000). Sound localization with eccentric head position. Behav. Brain Res. 108, 105–125. doi: 10.1016/S0166-4328(99)00141-2, PMID: 10701655

[ref105] Little A. D. Mershon D. H. Cox P. H. (1992). Spectral content as a cue to perceived auditory distance. Perception 21, 405–416. doi: 10.1068/p210405, PMID: 1437460

[ref106] Loomis J. M. Hebert C. Cicinelli J. G. (1990). Active localization of virtual sounds. J. Acoust. Soc. Am. 88, 1757–1764. doi: 10.1121/1.400250 2262632

[ref107] Loomis J. M. Klatzky R. L. Philbeck J. W. Golledge R. G. (1998). Assessing auditory distance perception using perceptually directed action. Percept. Psychophys. 60, 966–980. doi: 10.3758/BF03211932, PMID: 9718956

[ref9008] Macpherson E. A. Center W. (1994). On the role of head-related transfer function spectral notches in the judgement of sound source elevation. In: Proceedings of the 2nd International Conference on Auditory Display, 187–194.

[ref108] Macpherson E. A. Middlebrooks J. C. (2000). Localization of brief sounds: effects of level and background noise. J. Acoust. Soc. Am. 108, 1834–1849. doi: 10.1121/1.1310196, PMID: 11051510

[ref109] Majdak P. Baumgartner R. Jenny C. (2020). “Formation of three-dimensional auditory space” in The technology of binaural understanding. Modern acoustics and signal processing. eds. Blauert J. Braasch J. (Cham: Springer).

[ref110] Majdak P. Goupell M. J. Laback B. (2010). 3-D localization of virtual sound sources: effects of visual environment, pointing method, and training. Atten. Percept. Psychophys. 72, 454–469. doi: 10.3758/APP.72.2.454, PMID: 20139459 PMC2885955

[ref111] Mäkivirta A. Malinen M. Johansson J. Saari V. Karjalainen A. Vosough P. (2020). “Accuracy of photogrammetric extraction of the head and torso shape for personal acoustic HRTF modeling” in 148th audio engineering society international convention.

[ref112] Makous J. C. Middlebrooks J. C. (1990). Two-dimensional sound localization by human listeners. J. Acoust. Soc. Am. 87, 2188–2200. doi: 10.1121/1.399186 2348023

[ref113] Marmel F. Marrufo-Pérez M. I. Heeren J. Ewert S. Lopez-Poveda E. A. (2018). Effect of sound level on virtual and free-field localization of brief sounds in the anterior median plane. Hear. Res. 365, 28–35. doi: 10.1016/j.heares.2018.06.004, PMID: 29909353

[ref114] Mauermann M. Long G. R. Kollmeier B. (2004). Fine structure of hearing threshold and loudness perception. J. Acoust. Soc. Am. 116, 1066–1080. doi: 10.1121/1.1760106, PMID: 15376673

[ref115] McIntyre J. Stratta F. Droulez J. Lacquaniti F. (2000). Analysis of pointing errors reveals properties of data representations and coordinate transformations within the central nervous system. Neural Comput. 12, 2823–2855. doi: 10.1162/089976600300014746, PMID: 11112257

[ref116] Mendonça C. Campos G. Dias P. Vieira J. Ferreira J. P. Santos J. A. (2012). On the improvement of localization accuracy with non-individualized HRTF-based sounds. J. Audio Eng. Soc. 60, 821–830.

[ref117] Mershon D. H. King L. E. (1975). Intensity and reverberation as factors in the auditory perception of egocentric distance. Percept. Psychophys. 18, 409–415. doi: 10.3758/BF03204113

[ref118] Meshram A. Mehra R. Yang H. Dunn E. Franm J. M. Manocha D. (2014). P-HRTF: Efficient personalized HRTF computation for high-fidelity spatial sound. In 2014 IEEE International Symposium on Mixed and Augmented Reality (ISMAR), IEEE. 53–61.

[ref119] Miccini R. Spagnol S. (2020). HRTF individualization using deep learning. in proceedings -2020 IEEE conference on virtual reality and 3D user interfaces, VRW, IEEE. 390–395.

[ref120] Middlebrooks J. C. (1999a). Individual differences in external-ear transfer functions reduced by scaling in frequency. J. Acoust. Soc. Am. 106, 1480–1492. doi: 10.1121/1.427176, PMID: 10489705

[ref121] Middlebrooks J. C. (1999b). Virtual localization improved by scaling nonindividualized external-ear transfer functions in frequency. J. Acoust. Soc. Am. 106, 1493–1510. doi: 10.1121/1.427147, PMID: 10489706

[ref122] Middlebrooks J. C. Makous J. C. Green D. M. (1989). Directional sensitivity of sound-pressure levels in the human ear canal. J. Acoust. Soc. Am. 86, 89–108. doi: 10.1121/1.398224, PMID: 2754111

[ref123] Miller G. A. (1947). Sensitivity to changes in the intensity of white noise and its relation to masking and loudness. J. Acoust. Soc. Am. 19, 609–619. doi: 10.1121/1.1916528

[ref124] Mills A. W. (1958). On the minimum audible angle. J. Acoust. Soc. Am. 30, 237–246. doi: 10.1121/1.1909553

[ref125] Mills A. W. (1960). Lateralization of high-frequency tones. J. Acoust. Soc. Am. 32, 132–134. doi: 10.1121/1.1907864

[ref126] Mills A. W. Tobias J. V. (1972). Foundations of modern auditory theory. JV Tobias 2.

[ref127] Møller H. (1992). Fundamentals of binaural technology. Appl. Acoust. 36, 171–218. doi: 10.1016/0003-682X(92)90046-U, PMID: 29352772

[ref128] Møller H. Sørensen M. F. Jensen C. B. Hammershøi D. (1996). Binaural technique: do we need individual recordings? J. Audio Eng. Soc. 44, 451–469.

[ref129] Moore B. C. J. Glasberg B. R. (1996). A revision of Zwicker’s loudness model. Acta Acust. 82, 335–345.

[ref130] Neuhoff J. G. (2004). “Auditory motion and localization” in Ecological psychoacoustics (Brill: Elsevier), 87–111.

[ref131] Nilsson M. E. Schenkman B. N. (2016). Blind people are more sensitive than sighted people to binaural sound-location cues, particularly inter-aural level differences. Hear. Res. 332, 223–232. doi: 10.1016/j.heares.2015.09.012, PMID: 26433052

[ref132] Noble W. G. (1981). Earmuffs, exploratory head movements, and horizontal and vertical sound localization. J. Aud. Res. 21, 1–12, PMID: 7349864

[ref133] Nordmark J. O. (1976). Binaural time discrimination. J. Acoust. Soc. Am. 60, 870–880. doi: 10.1121/1.381167

[ref134] Oberem J. Richter J. G. Setzer D. Seibold J. Koch I. Fels J. (2020). Experiments on localization accuracy with non-individual and individual HRTFs comparing static and dynamic reproduction methods. bioRxiv.

[ref135] Ocklenburg S. Hirnstein M. Hausmann M. Lewald J. (2010). Auditory space perception in left- and right-handers. Brain Cogn. 72, 210–217. doi: 10.1016/j.bandc.2009.08.013, PMID: 19786316

[ref136] Odegaard B. Wozny D. R. Shams L. (2015). Biases in visual, auditory, and audiovisual perception of space. PLoS Comput. Biol. 11:e1004649. doi: 10.1371/journal.pcbi.1004649, PMID: 26646312 PMC4672909

[ref137] Oldfield S. R. Parker S. P. A. (1984). Acuity of sound localisation: a topography of auditory space. II. Pinna cues absent. i-Perception 13, 601–617. doi: 10.1068/p130601, PMID: 6535984

[ref138] Otte R. J. Agterberg M. J. H. Van Wanrooij M. M. Snik A. F. M. Van Opstal A. J. (2013). Age-related hearing loss and ear morphology affect vertical but not horizontal sound-localization performance. J. Assoc. Res. Otolaryngol. 14, 261–273. doi: 10.1007/s10162-012-0367-7, PMID: 23319012 PMC3660912

[ref139] Parise C. V. Spence C. Ernst M. O. (2012). When correlation implies causation in multisensory integration. Curr. Biol. 22, 46–49. doi: 10.1016/j.cub.2011.11.039, PMID: 22177899

[ref140] Parseihian G. Jouffrais C. Katz B. F. G. (2014). Reaching nearby sources: comparison between real and virtual sound and visual targets. Front. Neurosci. 8:269. doi: 10.3389/fnins.2014.00269, PMID: 25228855 PMC4151089

[ref141] Pastore M. T. Natale S. J. Yost W. A. Dorman M. F. (2018). Head movements allow listeners bilaterally implanted with cochlear implants to resolve front-back confusions. Ear Hear. 39, 1224–1231. doi: 10.1097/AUD.0000000000000581, PMID: 29664750 PMC6191386

[ref142] Pernaux J.-M. J. M. Emerit M. Nicol R. (2003). Perceptual evaluation of binaural sound synthesis: the problem of reporting localization judgments. AES 114th conv.

[ref143] Perrett S. Noble W. (1997a). The contribution of head motion cues to localization of low-pass noise. Percept. Psychophys. 59, 1018–1026. doi: 10.3758/BF03205517, PMID: 9360475

[ref144] Perrett S. Noble W. (1997b). The effect of head rotations on vertical plane sound localization. J. Acoust. Soc. Am. 102, 2325–2332. doi: 10.1121/1.419642, PMID: 9348691

[ref145] Perrott D. R. (1984). Concurrent minimum audible angle: a re-examination of the concept of auditory spatial acuity. J. Acoust. Soc. Am. 75, 1201–1206. doi: 10.1121/1.390771, PMID: 6725770

[ref146] Perrott D. R. Saberi K. (1990). Minimum audible angle thresholds for sources varying in both elevation and azimuth. J. Acoust. Soc. Am. 87, 1728–1731. doi: 10.1121/1.399421, PMID: 2341677

[ref147] Petersen J. (1990). Estimation of loudness and apparent distance of pure tones in a free field. Acta Acust. 70:5.

[ref148] Poirier-Quinot D. Katz B. F. G. (2018). “Impact of HRTF individualization on player performance in a VR shooter game II” in Proceedings of the AES international conference on Audio for Virtual and Augmented Reality.

[ref149] Pollack I. Rose M. (1967). Effect of head movement on the localization of sounds in the equatorial plane. Percept. Psychophys. 2, 591–596. doi: 10.3758/BF03210274

[ref150] Populin L. C. (2008). Human sound localization: measurements in untrained, head-unrestrained subjects using gaze as a pointer. Exp. Brain Res. 190, 11–30. doi: 10.1007/s00221-008-1445-2, PMID: 18575853 PMC3073845

[ref151] Pralong D. Carlile S. (1996). The role of individualized headphone calibration for the generation of high fidelity virtual auditory space. J. Acoust. Soc. Am. 100, 3785–3793. doi: 10.1121/1.417337, PMID: 8969480

[ref152] Rajendran V. G. Gamper H. (2019). Spectral manipulation improves elevation perception with non-individualized head-related transfer functions. J. Acoust. Soc. Am. 145, EL222–EL228. doi: 10.1121/1.5093641, PMID: 31067970

[ref153] Rakerd B. Vander Velde T. J. Hartmann W. M. (1998). Sound localization in the median sagittal plane by listeners with presbyacusis. J. Am. Acad. Audiol. 9, 466–479. 9865779

[ref154] Rayleigh L. (1907). XII. On our perception of sound direction. Philos. Mag. 13, 214–232. doi: 10.1080/14786440709463595

[ref155] Recanzone G. H. Makhamra S. D. D. R. Guard D. C. (1998). Comparison of relative and absolute sound localization ability in humans. J. Acoust. Soc. Am. 103, 1085–1097. doi: 10.1121/1.421222, PMID: 9479763

[ref156] Reed D. K. Maher R. C. (2009). “An investigation of early reflection’s effect on front-back localization in spatial audio” in In Audio Engineering Society Convention 127. Audio Engineering Society.

[ref157] Reinhardt-Rutland A. H. (1995). Increasing-and decreasing-loudness aftereffects: asymmetrical functions for absolute rate of sound level change in adapting stimulus. J. Gen. Psychol. 122, 187–193. doi: 10.1080/00221309.1995.9921231, PMID: 7790848

[ref158] Rhodes G. (1987). Auditory attention and the representation of spatial information. Percept. Psychophys. 42, 1–14. doi: 10.3758/BF03211508, PMID: 3658631

[ref159] Riesz R. R. (1932). A relationship between loudness and the minimum perceptible increment of intensity. J. Acoust. Soc. Am. 4:6. doi: 10.1121/1.1901961

[ref160] Risoud M. Hanson J. N. Gauvrit F. Renard C. Bonne N. X. Vincent C. (2020). Azimuthal sound source localization of various sound stimuli under different conditions. Eur. Ann. Otorhinolaryngol. Head Neck Dis. 137, 21–29. doi: 10.1016/j.anorl.2019.09.007 31582332

[ref161] Risoud M. Hanson J. N. Gauvrit F. Renard C. Lemesre P. E. Bonne N. X.. (2018). Sound source localization. Eur. Ann. Otorhinolaryngol. Head Neck Dis. 135, 259–264. doi: 10.1016/j.anorl.2018.04.009 29731298

[ref162] Robinson D. W. Dadson R. S. (1956). Equal-loudness relations, and threshold of hearing for pure tones. J. Acoust. Soc. Am. 28, 763–764. doi: 10.1121/1.1905030

[ref163] Röhl M. Uppenkamp S. (2012). Neural coding of sound intensity and loudness in the human auditory system. J. Assoc. Res. Otolaryngol. 13, 369–379. doi: 10.1007/s10162-012-0315-6 22354617 PMC3346895

[ref164] Ronsse L. M. Wang L. M. (2012). Effects of room size and reverberation, receiver location, and source rotation on acoustical metrics related to source localization. Acta Acust. United Acust. 98, 768–775. doi: 10.3813/AAA.918558

[ref9009] Rummukainen O. S. Schlecht S. J. Habets E. A. (2018). Self-translation induced minimum audible angle. J. Acoust. Soc. Am, 144, 340–345. 10.1121/1.5064957 30404470

[ref165] Rychtáriková M. van den Bogaert T. Vermeir G. Wouters J. (2011). Perceptual validation of virtual room acoustics: sound localisation and speech understanding. Appl. Acoust. 72, 196–204. doi: 10.1016/j.apacoust.2010.11.012

[ref166] Sandel T. T. Teas D. C. Feddersen W. E. Jeffress L. A. (1955). Localization of sound from single and paired sources. J. Acoust. Soc. Am. 27, 842–852. doi: 10.1121/1.1908052

[ref167] Sayers B. M. (1964). Acoustic-image lateralization judgments with binaural tones. J. Acoust. Soc. Am. 36, 923–926. doi: 10.1121/1.1919121

[ref168] Schoeffler M. Westphal S. Adami A. Bayerlein H. Herre J. (2014). Comparison of a 2D-and 3D-based graphical user Interface for localization listening tests. In proc. of the EAA joint symposium on Auralization and Ambisonics.

[ref169] Shaw E. A. G. (1974). Transformation of sound pressure level from the free field to the eardrum in the horizontal plane. J. Acoust. Soc. Am. 56, 1848–1861. doi: 10.1121/1.1903522, PMID: 4443484

[ref170] Sherlock L. G. P. Perry T. T. Brungart D. S. (2021). Evaluation of extended-wear hearing aids as a solution for intermittently noise-exposed listeners with hearing loss. Ear Hear. 42, 1544–1559. doi: 10.1097/AUD.0000000000001044, PMID: 33974779

[ref171] Simpson W. E. Stanton L. D. (1973). Head movement does not facilitate perception of the distance of a source of sound. Am. J. Psychol. 86, 151–159. doi: 10.2307/1421856 4742382

[ref172] Spagnol S. (2020). HRTF selection by anthropometric regression for improving horizontal localization accuracy. IEEE Signal Process. Lett. 27, 590–594. doi: 10.1109/LSP.2020.2983633

[ref173] Speigle J. M. Loomis J. M. (1993). Auditory distance perception by translating observers. In proceedings of 1993 IEEE research properties in virtual reality symposium, VRAIS 1993.

[ref9010] Spiousas I. Etchemendy P. E. Eguia M. C. Calcagno E. R. Abregú E. Vergara R. O. (2017). Sound spectrum influences auditory distance perception of sound sources located in a room environment. Front. Psychol, 8, 251475. 10.3389/fpsyg.2017.00969 PMC5479918 28690556

[ref174] Stevens S. S. (1955). The measurement of loudness. J. Acoust. Soc. Am. 27, 815–829. doi: 10.1121/1.1908048

[ref175] Stevens S. S. (1958). Problems and methods of psychophysics. Psychol. Bull. 55, 177–196. doi: 10.1037/h0044251, PMID: 13567963

[ref176] Stevens S. S. Guirao M. (1962). Loudness, reciprocality, and partition scales. J. Acoust. Soc. Am. 34, 1466–1471. doi: 10.1121/1.1918370

[ref177] Stevens S. S. Newman E. B. (1936). The localization of actual sources of sound. Am. J. Psychol. 48, 297–306. doi: 10.2307/1415748, PMID: 38596081

[ref178] Stevens S. S. Volkmann J. (1940). The relation of pitch to frequency: a revised scale. Am. J. Psychol. 53:329. doi: 10.2307/1417526, PMID: 18808425

[ref179] Stitt P. Picinali L. Katz B. F. G. (2019). Auditory accommodation to poorly matched non-individual spectral localization cues through active learning. Sci. Rep. 9:1063. doi: 10.1038/s41598-018-37873-0, PMID: 30705332 PMC6355836

[ref180] Tabry V. Zatorre R. J. Voss P. (2013). The influence of vision on sound localization abilities in both the horizontal and vertical planes. Front. Psychol. 4:932. doi: 10.3389/fpsyg.2013.00932, PMID: 24376430 PMC3860057

[ref181] Thavam S. Dietz M. (2019). Smallest perceivable interaural time differences. J. Acoust. Soc. Am. 145, 458–468. doi: 10.1121/1.5087566, PMID: 30710981

[ref182] Thurlow W. R. Mangels J. W. Runge P. S. (1967). Head movements during sound localization. J. Acoust. Soc. Am. 42, 489–493. doi: 10.1121/1.1910605, PMID: 6075942

[ref183] Thurlow W. R. Mergener J. R. (1970). Effect of stimulus duration on localization of direction noise stimuli. J. Speech Hear. Res. 13, 826–838. doi: 10.1044/jshr.1304.826, PMID: 5491357

[ref184] Trapeau R. Schönwiesner M. (2018). The encoding of sound source elevation in the human auditory cortex. J. Neurosci. 38, 3252–3264. doi: 10.1523/JNEUROSCI.2530-17.2018, PMID: 29507148 PMC6596065

[ref185] Van Wanrooij M. M. Van Opstal A. J. (2004). Contribution of head shadow and Pinna cues to chronic monaural sound localization. J. Neurosci. 24, 4163–4171. doi: 10.1523/JNEUROSCI.0048-04.2004, PMID: 15115811 PMC6729291

[ref186] Viaud-Delmon I. Warusfel O. (2014). From ear to body: the auditory-motor loop in spatial cognition. Front. Neurosci. 8:283. doi: 10.3389/fnins.2014.00283, PMID: 25249933 PMC4155796

[ref187] Vliegen J. Van Opstal A. J. (2004). The influence of duration and level on human sound localization. J. Acoust. Soc. Am. 115, 1705–1713. doi: 10.1121/1.1687423, PMID: 15101649

[ref188] von Békésy G. (1938). Über die Entstehung der Entfernungsempfindung beim Hören. Akust. Zeitschrift. 3, 21–31.

[ref189] Wallach H. (1940). The role of head movements and vestibular and visual cues in sound localization. J. Exp. Psychol. 27, 339–368. doi: 10.1037/h0054629

[ref190] Wang Y. (2007). On the cognitive processes of human perception with emotions, motivations, and attitudes. Int. J. Cogn. Informat. Nat. Intell. 1, 1–13. doi: 10.4018/jcini.2007100101

[ref191] Wang J. Lu X. Sang J. Cai J. Zheng C. (2022). Effects of stimulation position and frequency band on auditory spatial perception with bilateral bone conduction. Trends Hear. 26:233121652210971. doi: 10.1177/23312165221097196, PMID: 35491731 PMC9067062

[ref950] Warren R. M. (1958). A basis for judgments of sensory intensity. Am. J. Psychol. 71, 675–687. 13627276

[ref192] Warren R. Sersen E. Pores E. (1958). A basis for loudness-judgments. Am. J. Psychol. 71, 700–709., PMID: 13627278

[ref193] Wenzel E. M. Arruda M. Kistler D. J. Wightman F. L. (1993). Localization using nonindividualized head-related transfer functions. J. Acoust. Soc. Am. 94, 111–123. doi: 10.1121/1.407089 8354753

[ref194] Werner S. Klein F. Sporer T. (2016). “Adjustment of the direct-to-reverberant-energy-ratio to reach externalization within a binaural synthesis system” In Audio Engineering Society Conference: 2016 AES International Conference on Audio for Virtual and Augmented Reality. Audio Engineering Society.

[ref195] Wickens C. D. (1991). Processing resources in attention. Mult. Perform.

[ref196] Wightman F. L. Kistler D. J. (1989). Headphone simulation of free-field listening. II: psychophysical validation. J. Acoust. Soc. Am. 85, 868–878. doi: 10.1121/1.397558, PMID: 2926001

[ref197] Wightman F. L. Kistler D. J. (1992). The dominant role of low-frequency interaural time differences in sound localization. J. Acoust. Soc. Am. 91, 1648–1661. doi: 10.1121/1.402445, PMID: 1564201

[ref198] Wightman F. Kistler D. (1997). “Factors affecting the relative salience of sound localization cues” in Binaural and spatial hearing in real and virtual environments. eds. Gilkey R. Anderson T. R. (Psychology Press), 1–23.

[ref199] Wightman F. L. Kistler D. J. (1999). Resolution of front–back ambiguity in spatial hearing by listener and source movement. J. Acoust. Soc. Am. 105, 2841–2853. doi: 10.1121/1.426899, PMID: 10335634

[ref200] Withington D. (1999). “Localisable Alarms” in Human factors in auditory warnings. eds. Stanton N. A. Edworthy J. (Routledge: Ashgate Publishing Ltd).

[ref201] Yost W. A. (1981). Lateral position of sinusoids presented with interaural intensive and temporal differences. J. Acoust. Soc. Am. 70, 397–409. doi: 10.1121/1.386775

[ref202] Yost W. A. (2016). Sound source localization identification accuracy: level and duration dependencies. J. Acoust. Soc. Am. 140, EL14–EL19. doi: 10.1121/1.4954870, PMID: 27475204 PMC5848824

[ref203] Yost W. A. (2017). “History of sound source localization: 1850-1950” In Proceedings of Meetings on Acoustics (Vol. 30, No. 1). AIP (American Institute of Physics) Publishing.

[ref204] Yost W. A. Zhong X. (2014). Sound source localization identification accuracy: bandwidth dependencies. J. Acoust. Soc. Am. 136, 2737–2746. doi: 10.1121/1.4898045, PMID: 25373973

[ref205] Zahorik P. (2002). Assessing auditory distance perception using virtual acoustics. J. Acoust. Soc. Am. 111, 1832–1846. doi: 10.1121/1.1458027, PMID: 12002867

[ref206] Zahorik P. Brungart D. S. Bronkhorst A. W. (2005). Auditory distance perception in humans: a summary of past and present research. Acta Acust. United Acust. 91, 409–420.

[ref207] Zahorik P. Wightman F. L. (2001). Loudness constancy with varying sound source distance. Nat. Neurosci. 4, 78–83. doi: 10.1038/82931, PMID: 11135648

[ref208] Zhang M. Kennedy R. A. Abhayapala T. D. Zhang W. (2011). Statistical method to identify key anthropometric parameters in hrtf individualization. In 2011 joint workshop on hands-free speech communication and microphone arrays, HSCMA’11. IEEE.

[ref209] Zhong X. Yost W. A. (2017). How many images are in an auditory scene? J. Acoust. Soc. Am. 141:2882. doi: 10.1121/1.4981118 28464690 PMC6909977

[ref210] Zotkin D. N. Duraiswami R. Davis L. S. (2002). Creation of virtual auditory spaces. In ICASSP, IEEE international conference on acoustics, speech and signal processing-proceedings. (Vol. 2, pp. II-2113). IEEE.

[ref211] Zotkin D. N. Duraiswami R. Davis L. S. (2004). Rendering localized spatial audio in a virtual auditory space. IEEE Trans. Multimed. 6, 553–564. doi: 10.1109/TMM.2004.827516

[ref212] Zotkin D. Y. N. Hwang J. Duraiswaini R. Davis L. S. (2003). “HRTF personalization using anthropometric measurements” in IEEE workshop on applications of signal processing to audio and acoustics. (IEEE Cat. No. 03TH8684). (pp. 157-160). IEEE.

[ref213] Zwicker E. (1961). Subdivision of the audible frequency range into critical bands (Frequenzgruppen). J. Acoust. Soc. Am. 33:248. doi: 10.1121/1.1908630

[ref214] Zwislocki J. Feldman R. S. (1956). Just noticeable differences in dichotic phase. J. Acoust. Soc. Am. 28, 860–864. doi: 10.1121/1.1908495

